# Metabolomic Analysis of Cannabinoid and Essential Oil Profiles in Different Hemp (*Cannabis sativa* L.) Phenotypes

**DOI:** 10.3390/plants10050966

**Published:** 2021-05-12

**Authors:** Marjeta Eržen, Iztok J. Košir, Miha Ocvirk, Samo Kreft, Andreja Čerenak

**Affiliations:** 1 Slovenian Institute of Hop Research and Brewing, Cesta Žalskega tabora 2, 3310 Žalec, Slovenia; marjeta.erzen@ihps.si (M.E.); iztok.kosir@ihps.si (I.J.K.); miha.ocvirk@ihps.si (M.O.); 2 Faculty of Pharmacy, University of Ljubljana, Aškerčeva cesta 7, 1000 Ljubljana, Slovenia; samo.kreft@ffa.uni-lj.si

**Keywords:** *Cannabis sativa* L., Cannabaceae, cannabinoids, essential oils, terpenes, GC/FID, HPLC

## Abstract

Hemp (*Cannabis sativa* L.) cannabinoids and terpenoids have therapeutic effects on human and animal health. Cannabis plants can often have a relatively high heterogeneity, which leads to different phenotypes that have different chemical profiles despite being from the same variety. Little information exists about cannabinoid and terpenoid profiles in different hemp phenotypes within the same variety. For this study, 11 phenotypes from three different varieties (“Carmagnola” selected (CS), “Tiborszallasi” (TS), and “Finola” selection (FS)) were analyzed. The components of essential oil (29) were analyzed using gas chromatography with flame ionization detection (GC/FID), and 10 different cannabinoids of each phenotype were determined using high-performance liquid chromatography (HPLC). Principal component analysis (PCA) and analysis of variance (ANOVA) showed that according to the components of essential oil, FS and TS plants were more uniform than CS plants, where there were great differences between CI and CII phenotypes. The content of cannabinoid CBD-A was the highest in all four FS phenotypes. By comparing cannabinoid profiles, FS was clearly separated from TS and CS, while these two varieties were not clearly distinguishable. Phenotypes TV and CI had the highest total content of Δ-9-THC, while all phenotypes of FS had the highest total content of CBD. The highest total content of CBG was determined in phenotype CI. Obtained results are useful for the development of new supplementary ingredients, for different pharmacy treatments, and for further breeding purposes.

## 1. Introduction

Hemp (*Cannabis sativa* L.) originated from central Asia and has been used for human and animal food, as a source of fiber for ropes, and in medicine [1,2]. It contains more than 500 phytochemicals with many therapeutic purposes and has been used to treat epilepsy, Alzheimer’s disease, Parkinson’s disease, multiple sclerosis, pain and nausea in cancer patients, diabetes, and eating disorders [3].

The most well-known phytochemicals are secondary metabolites, such as cannabinoids and terpenoids [4]. More than 150 cannabinoids have already been identified in hemp [5]. The most active and studied compounds are Δ-9-tetrahydrocannabinol (Δ-9-THC), cannabidiol (CBD), cannabigerol (CBG), cannabichromene (CBC), and their carboxylated forms [3]. Terpenoids in essential oil are divided into two main groups, monoterpenes and sesquiterpenes, which are responsible for hemp fragrance and flavor and also contribute to therapeutic effects. There are generally fewer sesquiterpenes than monoterpenes detected in hemp flowers. The highest content of cannabinoids and terpenoids is found in the glandular trichomes on bracts [6].

Precursors for cannabinoids have two biosynthetic pathways. The polyketide pathway leads to olivetolic acid (OLA), and the plastidial 2-C-methyl-D-erytritol 4-phosphate (MEP) pathway leads to geranyl diphosphate (GPP). Precursors OLA and GPP form cannabigerolic acid (CBG-A), which is a precursor for different cannabinoids, as well as THC-A, CBD-A, and CBC-A [7]. Terpenoids are composed of isoprene units. Similar to cannabinoids, terpenoids also have different biosynthetic pathways. Sesquiterpenes and triterpenes are formed from the cytosolic mevalonic acid (MVA) pathway, while monoterpenes, diterpenes, and tetraterpenes are formed via the plastid-localized (MEP) pathway. Subsequently, precursors of sesquiterpene farnesyl diphosphate (FPP) and monoterpene geranyl diphosphate are formed [7].

Terpenoids and cannabinoids may have a synergistic effect on human and animal health [8]. An example of the positive effects of the combined use of cannabinoids and terpenoids is acne therapy, in which CBD, limonene, linalool, and pinene are involved. Cannabinoids and terpenoids, such as CBG and pinene, also have a combined effect on MRSA (methicillin-resistant *Staphylococcus aureus*) [9,10]. However, the issue of synergy remains controversial and needs further investigation.

According to the chemical composition, there are five major hemp chemotypes. Small and Beckstead [11] determined three chemotypes: chemotype I with a THC content higher than 0.3% and CBD content lower than 0.5%, chemotype II (intermediate type) with a THC and CBD ratio that is roughly equal, and chemotype III with a higher CBD content than 0.5% and THC content lower than 0.3% of the flower dry matter. Later, Fournier et al. [12] determined two other chemotypes: chemotype IV, with a prevalence of CBG higher than 0.3% of the flower dry matter, and chemotype V, with an undetectable content of cannabinoids. Numerous scientists studied species and subspecies of *Cannabis* [13]. In general, it is known that hemp and marijuana differ based on THC content. Hemp is supposed to have THC content below 0.2–1%, which depends on the legislation of different countries, while marijuana could reach THC content up to 20 to 30% in dry inflorescences [14]. In 2015, Sawler et al. [15] determined that hemp and marijuana significantly differ at the genome level, that different marijuana types are often not genetically close, and that THC is not related to the genetic distinction between hemp and marijuana. Hemp has been used for food and fibers, while marijuana was mostly used in traditional medicine [16]. However, marijuana was prohibited and criminalized all around the world due to the psychoactive nature of THC. In 1988 United Nations Convention against Illicit Traffic in Narcotic Drugs and Psychotropic Substances prohibited the use, production, and cultivation of *Cannabis*, which was recognized as a narcotic drug with psychotropic compounds, still posing a major problem in the legalization of *Cannabis* with higher THC content [17]. Due to many different research studies based on the positive effects of *Cannabis* on numerous diseases, more and more countries are slowly releasing their legislation in favor of growing *Cannabis* for medical and scientific purposes in restricted area.

In *Cannabis,* the varieties (strains) are often not fully inbred; therefore, they have a relatively high level of heterogeneity and instability, compared to other crops. Different phenotypes can be found within one variety. One of the major breeding challenges is that *Cannabis* plants are mostly dioecious, and they cannot pollinate themselves; hence, outcrossing commonly occurs [18]. This paper aimed to clarify the distinction between three different hemp varieties and their 11 phenotypes according to an analysis of cannabinoids and terpenoids. Varieties Carmagnola selected, Tiborszallasi, and Finola selection were chosen because of the expressed interest of our partners from the industry. All of them are registered on the EU variety list and are grown as out-growing varieties. Together, we found out that different phenotypes could be detected within them, and we supposed that they could express different chemical profiles as well, with a further different application in pharmacy.

The objective was to establish a connection between the chemical composition and morphological characteristics of hemp plants and to identify phenotypes with an interesting ratio between cannabinoids for further pharmaceutical applications.

## 2. Results and Discussion

### 2.1. Differentiation of Phenotypes according to Visual Traits

In total, 11 different phenotypes were selected according to visual traits observed within varieties that were compared with each other, and also reference types [19,20,21] (certified types from breeders) were added for each variety (Table 1). However, this is a first preliminary comparison between different phenotypes within mentioned varieties. Photos of all 11 phenotypes are presented in Figures S3–S14. According to the Slovenian Ministry of Agriculture, Forestry, and Food in 2020, there was 6.67 ha of Carmagnola, 0.86 ha of Tiborszallasi, and 19.77 ha of Finola. 

Five plants with the same observed traits within one phenotype were labeled and separately sampled. Hemp is an open-pollinated plant and, therefore, also more prone to nonuniformity. Pollen can disperse a few kilometers in relation to the wind direction [22], which could be one of the reasons for higher heterogeneity in *Cannabis* varieties. Additionally, newly bred populations and marijuana populations are more uniform and can be easily grouped by their desirable traits, such as high THC/CBD level, high limonene, or other terpenoid levels [15].

### 2.2. Chemical Analysis of the Essential Oil of Hemp (Cannabis sativa L.)

Briefly, 11 phenotypes from “Carmagnola” selected (CS), “Tiborszallasi” (TS), and “Finola” selection (FS) contained 0.09–3.38 mL of essential oil per 100 g of air-dried flower (1.34 mL/100 g, on average). FS achieved the highest average content of essential oil (2.81 mL/100 g air-dried flower), compared to CS (0.38 mL/100 g air-dried flower) and TS (0.54 mL/100 g air-dried flower). The greatest relative difference between phenotypes within varieties was between CS phenotypes (*p* < 0.0001) in relation to essential oil. CI achieved 0.23 mL/100 g of air-dried flower, while CII achieved 0.53 mL/100 g air dry flower of essential oil. When comparing monoecious and dioecious varieties, Bertoli et al. [6] discovered a higher content of essential oil in dioecious plants. Nissen et al. [23] also reported about low content of essential oil in the CS variety. Significant differences have been recorded between different hemp varieties according to essential oil and cannabinoid content in previous studies as well [24,25].

As expected, the most abundant terpenoids were myrcene, β-caryophyllene, α-pinene, and α-humulene in all three varieties [3,6,23]. The proportion of the 10 main components of essential oil were compared with analysis of variance (ANOVA) and presented in superscripts in Table 2 and in Table S1, in which all 29 analyzed components are presented. According to ANOVA, phenotypes CI and CII showed significant differences based on α-pinene, β-pinene, 3-carene, terpinolene, β-caryophyllene, α-humulene, caryophyllene oxide, β-eudesmol, and phytol. The CII phenotype had significantly higher contents of all main monoterpenes (α-pinene, β-pinene, 3-carene, and terpinolene) than phenotype CI, while the contents of all main sesquiterpenes (β-caryophyllene, α-humulene, caryophyllene oxide, β-eudesmol) and phytol were significantly higher in the CI phenotype. When comparing phenotypes of TS, differences were shown in caryophyllene oxide. Terpinolene and α-terpinene were found at different concentrations in FS phenotypes. As is evident from Table 2, the phenotypes of FS had very high proportions of limonene (4.1–5.2%), in comparison to CS and TS phenotypes, which is of great value since FS phenotypes also reached the highest content of essential oil. The lowest proportion of limonene was in the TI phenotype (0.6%). Phenotypes of FS had the highest proportions of α-terpineol, β-eudesmol, and α-bisabolol and very low proportions of caryophyllene, oxide, and undetachable proportion of phytol, cis-nerolidol, etc. The highest proportion of α-pinene was in all TS phenotypes, especially in TIV (11.9%), and in phenotype CII (11.9%). A distinctly lower proportion was identified in FIV (0.7%). The highest proportion of myrcene was also observed in the five TS phenotypes; TI had the highest proportion among the TS phenotypes (29.9%). The highest proportion of, β-pinene, 3-carene, γ-terpinene, terpinolene, borneol, and menthol occurred in CII among all phenotypes, while the highest proportion of α-cedrene, β-caryophyllene, α-humulene, cis-nerolidol, geranyl isobutyrate, caryophyllene oxide, β-eudesmol, and phytol occurred in CI (almost all components of sesquiterpenes). However, the CS variety also had the lowest amount of essential oil compared to other investigated varieties. FIII had the highest proportion of α-terpinene (0.6%) and fenchone (0.2%), and TI had the highest proportion of p-cymene (0.1%). The highest proportion of borneol (0.2%) and geranyl acetate (0.3%) was in FIV. FII contained the most α-terpineol (1.0%). Terpenoids p-cymene, camphor, isoborneol, β-citronellol, and neryl acetate had contents lower than 0.1% or were undetectable. 

Terpenoids have a great impact on human health [26]. Almost all listed components have anti-inflammatory effects, including myrcene, β-caryophyllene, caryophyllene oxide, humulene, α-pinene, linalool, limonene, terpinolene, γ-terpinene, nerolidol, borneol, fenchone, and β-eudesmol. Some components have positive impacts on cancer and tumor treatments, such as β-caryophyllene, which also has a synergistic anticancer effect with humulene. Iso-caryophyllene, humulene, α-pinene, and linalool have antitumor activity [27]. All phenotypes of CS and TS had high proportions of caryophyllene oxide, which has a positive effect on type 1 and type 2 diabetes, cardiovascular diseases, and hypertension [27,28]. CII had the highest proportion of β-pinene, which has an important role in the regulation of diabetes, cancer, obesity, and other chronic diseases. Myrcene, terpinolene, linalool, and nerolidol have sedative effects. Humulene and limonene treat depression, and humulene has also been used in traditional medicine for treating insomnia, anxiety, delirium, and depression. With a significantly higher proportion of α-humulene, the CI phenotype could be used in further pharmaceutical research, especially if the total amount of essential oil could be increased in breeding procedures or affected by the environment. This phenotype also had a significantly higher proportion of β-eudesmol, which stimulates appetite [27].

The total amount of detected monoterpenes was higher than that of sesquiterpenes in all three varieties [6,28]. Comparing the investigated varieties, the highest monoterpene content (44.89%) occurred in TS, while CS varieties had the highest amount of sesquiterpenes (28.67%). In the group of monoterpenes belong compounds α-pinene, camphene, β-pinene, myrcene, 3-carene, α-terpinene, p-cimene, limonene, γ-terpinene, fenchone, terpinolene, linalool, camphor, isoborneol, borneol, menthol, α-terpineol, β-citronellol, neryl acetate, and geranyl acetate, while in the group of sesquiterpenes belong compounds α-cedrene, β-caryophyllene, α-humulene, cis-nerolidol, geranyl isobutyrate, caryophyllene oxide, β-eudesmol, α-bisabolol, and phytol. 

**Figure 1:**
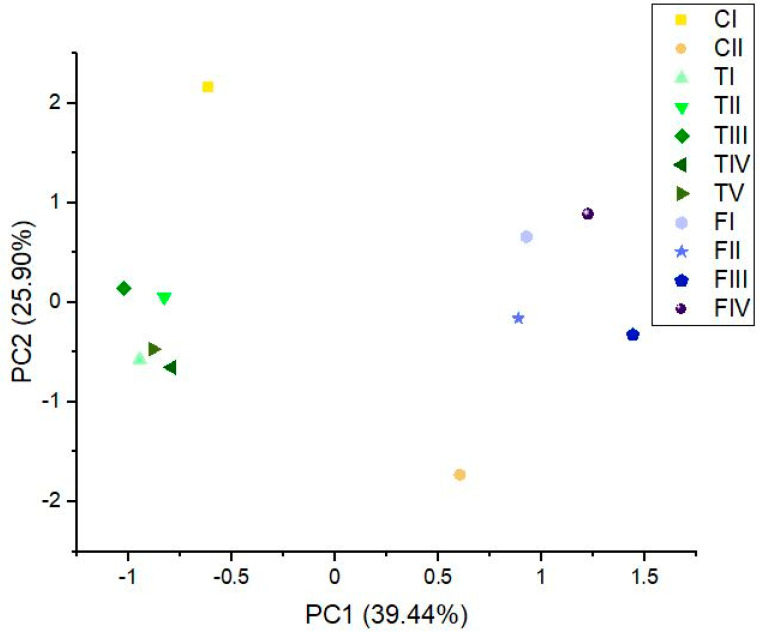
Principal component analysis (PCA) plots for phenotype averages according to essential oil components made on the first two PC scores (PC1 explained 39.44%, and PC2 explained 25.90%), with a total variance of 65.34%.

A difference between varieties was observed when the average of essential oil components in individual phenotypes was compared (Figure 1). Principal component (PC)1 explained 39.44%, and PC2 explained 25.90% of the variance. Phenotypes of FS and TS were clustered together, while phenotypes of CS were separated. Considering the average amount of compounds in essential oil phenotypes within FS and TS, they were more uniform than CS phenotypes. More detailed PCA plots with all analyzed variables are presented in Figure S1. 

**Figure 2:**
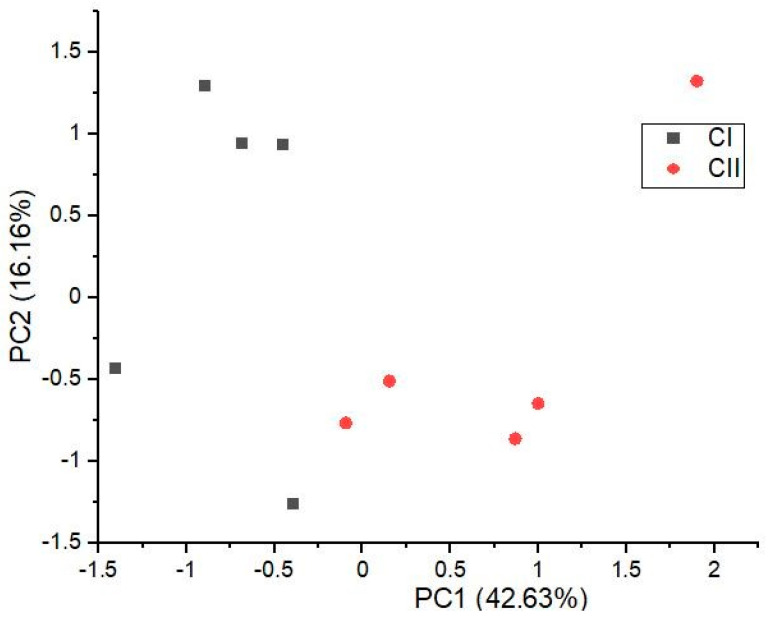
Principal component analysis (PCA) plots for phenotypes of the Carmagnola selected variety, according to components of essential oil made on the first two PC scores (PC1 explained 42.63%, and PC2 explained 16.16%), with a total variance of 58.79%.

According to all analyzed components of essential oil, each individual variety of CS, TS, and FS was analyzed separately. Significant differences between both phenotypes of CS that were found by ANOVA can clearly also be observed in the PCA (Figure 2). TS and FS phenotypes were not distinguishable, which means that these two varieties were more uniform than CS. 

### 2.3. Chemical Analysis of Cannabinoids in Hemp (Cannabis sativa L.) Phenotypes

Regarding the analysis of cannabinoids, 10 different cannabinoids were identified in the 11 included phenotypes using high-performance liquid chromatography (HPLC) (Table 3). There was a significant difference in CBD-A and CBG-A cannabinoids between the CI and CII phenotypes. CII was more related to TS phenotypes. There were also significant differences in cannabinoids CBD and CBC between FS phenotypes and all other phenotypes.

CBD-A represented the highest proportion of cannabinoids in all included phenotypes, with the highest content in the four FS phenotypes (6.36–6.59%). Compared to the other phenotypes, the highest proportion of CBG-A was in phenotype CI (1.62%), followed by all FS phenotypes. Phenotype TV and CI had the highest proportion of Δ-9-THC-A, while the highest proportions of Δ-9-THC were in the phenotypes of FS (0.08–0.11%). The lowest proportion of Δ-9-THC-A and Δ-9-THC were in phenotypes TII and TIV. The CBN values were less than 0.04%, and Δ-8-THC could not be detected in any sample. Aizpurua-Olaizola et al. [29] also determined the highest proportion of Δ-9-THC-A, CBD-A, and CBG-A in clones of mother plants from chemotypes I, II, and III in an unknown variety. Glivar et al. [30] analyzed the cannabinoid content in 15 different hemp varieties; when comparing proportions of CBD-A, CBD, THC, and Δ-9-THC-A in bracts of TS, their values were higher than those found in our samples, while other proportions were comparable with our data. In CS, there were higher proportions of all components, compared to the components in our CS variety, except for Δ-9-THC-A, which was higher in our CS samples. [30].

As mentioned before, Small and Beckstead [11] and Fournier et al. [12] already determined five major chemotypes based on the chemical profile of different hemp plants, whether it is marijuana or hemp type, while our classification further divides varieties in different phenotypes. Nevertheless, based on almost the same ratio between CBD and Δ -9-THC and CBD-A and Δ-9-THC-A, phenotypes CI and TV belong to chemotype II, while all other phenotypes were classified as chemotype III due to a high CBD-A and low Δ-9-THC-A content. None of the phenotypes were chemotype I, which was expected since the investigation included hemp and not marijuana varieties. 

**Figure 3:**
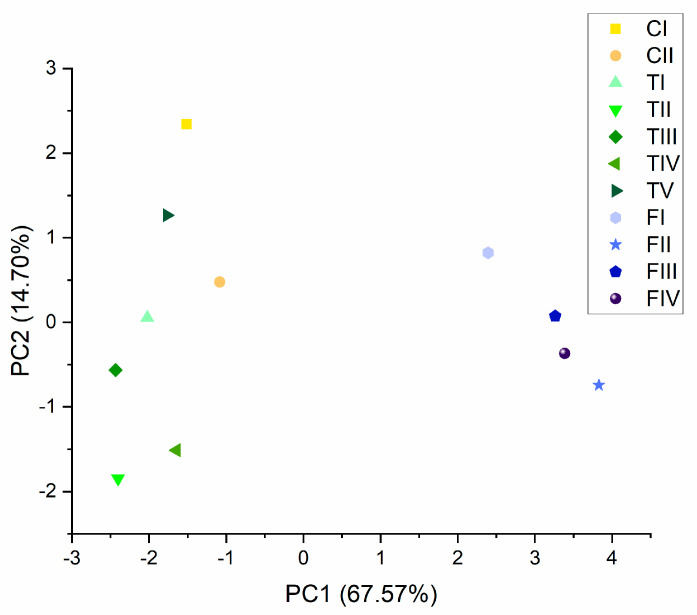
Principal component analysis (PCA) plot averages of analyzed cannabinoids for included phenotypes made on the first two PC scores (PC1 explained 67.57%, and PC2 explained 14.70%), with a total variance of 82.27%.

PCA plots for the averages of included phenotypes in all three varieties were performed according to cannabinoid content. PC1 explained 67.57%, and PC2 explained 15.70% of the variance seen in cannabinoid content. More detailed PCA plots with all analyzed variables are presented in Figure S2. There was a considerable difference between the phenotypes of FS compared to CS and TS, but we could not completely differentiate between varieties CS and TS. Nevertheless, there were greater differences between TS and CS phenotypes than between phenotypes within FS. The results are presented in Figure 3. Greater differences were observed based on essential oil proportions than proportions of cannabinoid content. 

**Figure 4:**
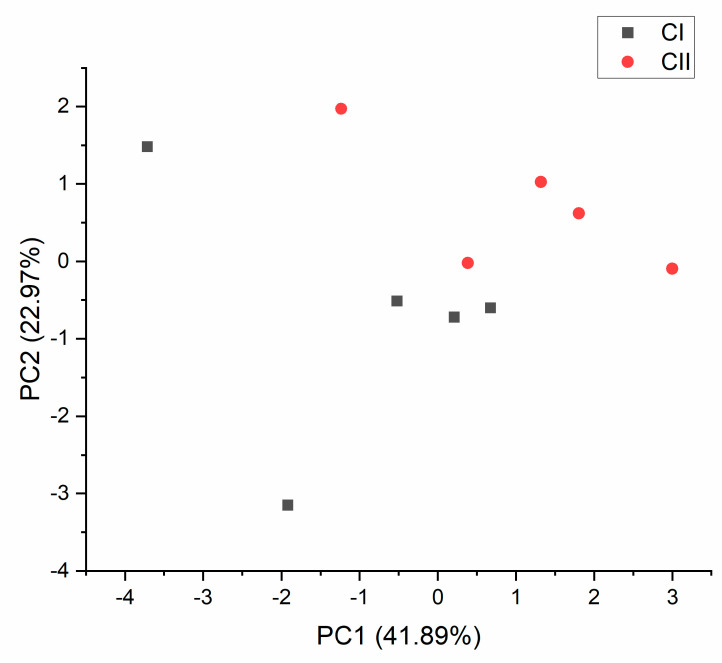
Principal component analysis (PCA) plots of cannabinoid content for the Carmagnola selected variety without Δ-8-THC, made on the first two PC scores (PC1 explained 41.89%, and PC2 explained 22.97%), with a total variance of 64.86%.

Each variety was analyzed separately to identify any differences between phenotypes within varieties. As with the essential oil components, differences in cannabinoids between CS phenotypes (CI and CII) were detected, while there were no differences between the five TS and four FS phenotypes. An additional PCA was performed for the CS variety, and clear differences between the two phenotypes were observed (Figure 4). PC1 explained 41.89%, and PC2 explained 22.97% of the variance in cannabinoid content. 

Aizpurua-Olaizola et al. [29] discovered differences between chemotypes and correlations with certain terpenoids and cannabinoids in different chemotypes. In that research, clones of different chemotypes were used. They discovered a higher similarity between chemotypes II and III than chemotype I. In our research, there were no evident differences between these two chemotypes. Terpenoids, such as γ-selinene, β-selinene, α-gurgujene, γ-elemene, selina-3,7(11)-diene, and β-curcumene, had a higher correlation with THC-A and a negative correlation with CBD-A in chemotype I, while terpenoids β-eudesmol, γ-eudesmol, guaiol, α-bisabolene, α-bisabolol, and eucalyptol had a higher correlation with CBD-A and negative correlation with THC-A in chemotype III [29]. However, Hillig [31] reported that the components β-eudesmol, γ-eudesmol, and guaiol were characteristic of *C. indica,* which has a higher level of Δ-9-THC. In our case, the CI phenotype had the highest relative level of β-eudesmol and a high level of Δ-9-THC-A. When comparing phenotypes of each variety separately, only CS phenotypes showed significant differences in CBD-A and CBG-A, while phenotypes of TS and FS were not distinguishable, except FI and FIV, where separation from other phenotypes within the variety was evident in PCA analysis. This also indicated that TS and FS varieties were more uniform than CS, although CS is a variety selected from Carmagnola, which is the oldest hemp landrace grown in Italy and has been used as a breeding parent for many new varieties [18].

### 2.4. Chemical Analysis of Both Cannabinoids and Essential Oil in Hemp (Cannabis sativa L.) Phenotypes

Due to the positive effects of *C. sativa* on human health, it is slowly gaining acceptance as a medicine. Due to the human endocannabinoid system, cannabinoids bind to different receptors through which they act on different diseases [7]. Numerous research studies were conducted on the medicinal use of *Cannabis.* Ware et al. [32] described 30 symptoms and diseases for which cannabis is used and most commonly reported. The most studied are multiple sclerosis, neuropathy, chronic pain, depression, arthritis, migraine, allergy, spinal pain asthma, and weight loss. Very important clinical studies were conducted also on treatments of several cancers, Alzheimer’s disease, diabetes, epilepsy, Huntington’s diseases, Tourette’s syndrome, and also for treating HIV/AIDS [29]. The benefits of using industrial hemp as medicine are in the low content of THC (lower than 0.3–1%), having no value as a recreational drug. Hemp with high CBD or other cannabinoid content, together with low THC content, could be available for general use without medical prescription and, on the other hand, farmers could grow industrial hemp for medicinal purposes without any special restrictions due to THC legislation. Consumption of hemp seeds has also positive effects on human health, especially because of the content of omega-3 and omega-6 fatty acids. Seeds contain also proteins, carbohydrates, oil and fibers, and minerals [33]. 

According to our results, specific phenotypes can be used for further breeding purposes with specific cannabinoid and/or terpenoid content, which could be used as supplementary ingredients or in pharmacy. Lewis et al. [34] reported different chemovars of *Cannabis* with desirable cannabinoid and terpenoid profiles. Comparing our results with their research, the CII and TIV phenotypes had a high proportion of α-pinene and a high proportion of CBD-A, which could be of interest due to their ability to improve learning and memory and as a modulator of THC overdose. TIV also had the most favorable ratio between total THC:CBD and high CBD content and Δ-9-THC under 0.2%, which is the limit for varieties registered on the EU variety list. This phenotype could be very interesting in the pharmaceutical industry for the production of CBD. Due to its low THC content, it could also be grown outdoors by farmers. Phenotype TV had a high proportion of α-pinene, a relatively high proportion of myrcene, and a THC-A and CBD-A ratio around 1:2. Myrcene is responsible for the sedative effects of *Cannabis,* and together with THC, it causes the “couch-lock” effect. All FS phenotypes had the highest amount of essential oil and cannabinoids, with especially high CBD, limonene, β-caryophyllene, and α-humulene proportions, which relieves pain and inflammation and treats addiction [34]. In the past, cannabis inflorescences have been used in traditional medicine for the treatment of various diseases, such as acute pain, wound healing, insomnia, cough, and mania, which are also the subject of modern medicine studies related to cannabinoids [35].

**Figure 5:**
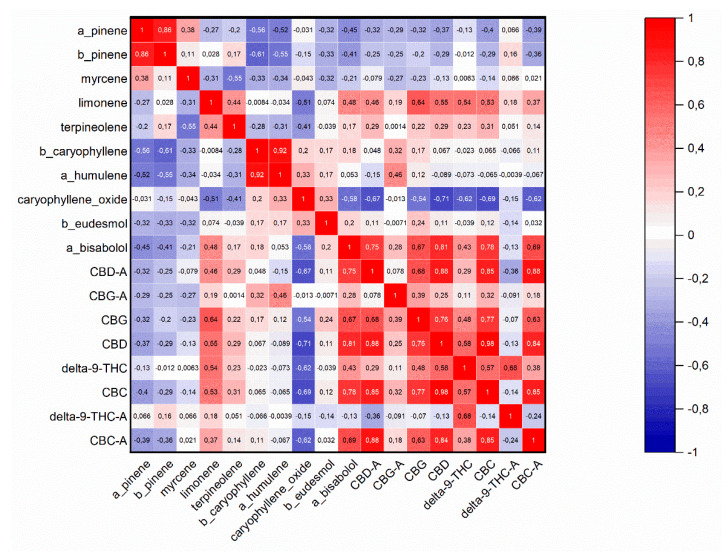
Pearson’s correlation coefficient of all three varieties and all phenotypes based on main terpenoids and cannabinoids.

Figure 5 explains Pearson’s correlation coefficient of all three varieties and their phenotypes based on the main terpenoids and cannabinoids. Only a correlation coefficient higher than 0.7 was considered a significant correlation. As seen in Figure 5, α-pinene and β-pinene had a strong positive correlation. Cannabinoid CBD-A had a strong positive correlation with sesquiterpene α-bisabolol and cannabinoids CBD, CBC, and CBC-A. Additionally, CBG showed a strong positive correlation with CBD and CBC. CBD had a negative correlation with caryophyllene oxide. Conversely, there was a positive correlation between CBG and cannabinoids CBD, CBC, and CBC-A. CBC had a strong positive correlation with α-bisabolol and CBC-A.

Our research was based on outdoor hemp cultivation. Industrial hemp is typically cultivated outside, where growing conditions are different from indoor cultivation. Therefore, the results of outdoor cultivation can vary from those of hemp cultivated indoors, where plants are exposed to more uniform conditions. In indoor cultivation, growing temperatures, day length, light, fertilization, irrigation, pest control, and harvest time are tightly determined to be optimal for growth [36]. Plants cultivated indoors are more stable and suitable for reproduction than plants grown outdoors [16]. However, our purpose was to identify the most suitable phenotypes that could be grown outdoors with appropriate technology and contain a high cannabinoid and terpenoid content [36].

## 3. Materials and Methods

### 3.1. Plant Material

Three different dioecious hemp (*Cannabis sativa* L.) varieties (Cannabaceae), Carmagnola selected (CS), Tiborszallasi (TS), and Finola selection (FS), were grown in 2019. Two different phenotypes of CS (CI, CII), five phenotypes of TS (TI, TII, TIII, TIV, TV), and four phenotypes of FS (FI, FII, FIII, FIV) were selected according to visual traits (habitus, leaf size, length and compactness of the inflorescence, color of leaf petioles, and internode length) (Table 1). Plants were observed 1–2 times weekly throughout the whole growing season, and final phenotypes were determined at the end of maturity. The inflorescences of 10 plants of each phenotype were separately sampled according to their aforementioned morphological characteristics at their optimal maturity time. For chemical analysis, the inflorescences of five plants per phenotype were used. Plants began flowering in the middle of August and were sampled and harvested at the end of September when most of the pistils turned brown. Male plants were removed from the crop before flowering to prevent female pollination and seed development. The lowest average temperatures were in the middle of May (11.4 °C) and the highest temperatures were in June (22.7 °C). Temperatures were higher than usual except in May when temperatures were lower than usual. From June to September, there was more precipitation than in 30 years on average. The soil was fertilized with organic fertilizer before sowing. No irrigation and no pest control were used. Weeds were controlled only mechanically twice at the beginning of growing. 

### 3.2. Standards and Reagents

The following terpenoid standards were purchased from Supelco: α-pinene, camphene, β-pinene, myrcene, 3-carene, α-terpinene, p-cymene, limonene, γ-terpinene, fenchone, terpinolene, linalool, camphor, isoborneol, borneol, menthol, α-terpineol, β-citronellol, neryl acetate, geranyl acetate, α-cedrene, β-caryophyllene, α-humulene, cis-nerolidol, geranyl isobutyrate, caryophyllene oxide, β-eudesmol, α-bisabolol, and phytol. Cannabinoid standards were purchased from Sigma Aldrich (1 mg/mL): cannabidiolic acid (CBD-A), cannabigerolic acid (CBG-A), cannabigerol (CBG), cannabidiol (CBD), cannabinol (CBN), Δ-9-tetrahydrocannabinol (Δ-9-THC), Δ-8-tetrahydrocannabinol (Δ-8-THC), cannabichromene (CBC), Δ-9-tetrahydrocannabinolic acid (Δ-9-THC-A), and cannabichromenic acid (CBC-A). Acid standards were dissolved in acetonitrile, and nonacid standards were dissolved in methanol. Hexane, ammonium format, and acetonitrile were purchased from Sigma Aldrich, methanol was purchased from J. T. Baker and formic acid was purchased from Scharlau. Chemical structures of essential oils and cannabinoids are presented in Supplementary Materials [37,38,39].

### 3.3. Sample Preparation

Harvested inflorescences without stems and the larger leaves were dried in the drier at 30 °C immediately after sample collection. Inflorescences were ground (30–50 g) and stored in the refrigerator at −20 °C until analysis. For gas chromatography with flame ionization detection (GC–FID), dried samples (27–30 g) of the 11 different phenotypes were steam distilled (3 h, 1 L distilled water) by a Clevenger apparatus. Each plant within each phenotype was analyzed separately with five different plants per phenotype. The collected oil was diluted with 1 mL of hexane and separated with gas chromatography (GC) analysis. For HPLC analysis, ground flowers (200 ± 9 mg) of 11 different phenotypes were weighed in 50 mL centrifuge tubes, and 25.0 mL of 80% methanol was added before vortexing for 30 s. Extraction was performed by sonicating in a bath for 15 min and vortexed every 5 min [40]. 

### 3.4. GC–FID Analysis

An Agilent GC 6890 (Agilent) with an HP 1 capillary column (Crosslinkeed Methyl Silicone Gum) of 2.5 mm × 0.2 mm × 0.1 mm (Agilent, Santa Clara, CA, USA) was used. The carrier gas flow (Helium 5.0) was set to 0.5 mL/min. One µL of the solution was injected into the injector port at a temperature of 180 °C. The oven temperature program was 3.5 min at 60 °C, 3.5 °C/min to 155 °C, and 30 °C/min to 300 °C. Detection was carried out on a flame ionization detector set at 300 °C with a 42.47 min runtime. Identification was made with ChemStation software for GC.

### 3.5. HPLC Analysis

For HPLC, an Agilent 1200 (Agilent, Santa Clara, CA, USA) was used. Separation was achieved on a Raptor ARC-18 (octadecylsilane) with a 2.7 µm, 150 mm × 4.6 mm ID (Restek, Bellefonte, PA, USA) column. Mobile phase A was composed of 5 mM ammonium formate and 0.1% formic acid in deionized water with a final pH of 3.1. Mobile phase B was composed of acetonitrile and 0.1% formic acid. 25% mobile phase A, and 75% mobile phase B. The flow rate was 1.5 mL/min, and an isocratic elution was in use. A calibration curve for each standard ranging from 5 to 250 µg/mL was prepared with correlation coefficients higher than 0.9975. The extracts were filtered through a disposable syringe with a PTFE filter (0.45 µm). The injection volume was 5 µL. Detection took place at 228 nm with a 15 min run time. Identification was carried out with ChemStation for LC. 

### 3.6. Statistical Analyses

Principal component analysis was performed using OriginPro 2021. Data were plotted based on the first two components. Multifactor ANOVA with Duncan test at a 95% confidential interval was performed in Statgraphics Centurion Software. Eleven phenotypes were compared according to the components of essential oil and cannabinoids, which were selected according to morphological differences between phenotypes. Means and standard deviations were calculated in Microsoft Excel.

## 4. Conclusions

In conclusion, the purpose of our study was to compare the composition of essential oil and cannabinoids in 11 hemp phenotypes from three varieties based on morphological distinctness. To our knowledge, this is the first research to study differences in cannabinoid and essential oil content in various phenotypes within different varieties of hemp. Our study showed that based on the content of cannabinoids and essential oil, FS significantly and positively differed from CS and TS varieties, making it important for the hemp industry. Based on PCA analysis, there were no evident differences between phenotypes in FS and TS within each analyzed individual variety, while the two CS phenotypes were distinctive. By comparing the average content of the detected compounds in essential oil and cannabinoids between individual phenotypes of all three varieties, differences were determined. Among all analyzed phenotypes, CI, TII, TIV, and TV showed an interesting ratio between THC and CBD for further use in the pharmaceutical industry and breeding. CI and TV had a similar ratio of THC and CBD, while TII and TIV had a very high level of CBD, which may be useful in the pharmaceutical industry for epilepsy treatment [41]. Additionally, the phenotypes of FS could be interesting due to a high CBD content, which could be extracted. When comparing phenotypes, according to ANOVA, significant differences in individual components of essential oil and cannabinoids were shown, potentiating the use of individual phenotypes in specialized breeding programs or disease treatments. Cannabinoids and terpenoids may have synergistic effects on different disease symptoms. When breeding or growing selected phenotypes, the plant varieties will be more homogenous regarding their chemical composition and, consequently, more interesting for pharmaceutical use. The cultivation of *C. sativa* with a low content of Δ-9-THC gives farmers an opportunity to cultivate hemp outdoor under controlled conditions for pharmaceutical purposes.

## Figures and Tables

**Table 1:** The 11 different phenotypes (CI, CII, TI, TII, TIII, TIV, TV, FI, FII, FIII, FIV) that are defined by 6 different visual traits; size, color, leaf size, inflorescences, anthocyanin coloration of leaf petiole, and branching. For each variety, reference types are added.

Phenotype	Size	Color	Leaf Size	Inflorescences	Anthocyanin Coloration of Leaf Petiole	Branching	Remarks
*‘Carmagnola selected’*							
CI	Tall	Light	Large	Small	No	***	
CII	Tall	Dark	Small	Small	Yes	*****	
Reference type	Tall	Dark	Medium	-	Medium	-	
*‘Tiborszallasi’*							
TI	Tall	Medium	Medium	Small	No	*****	
TII	Medium	Dark	Medium	Medium	Yes	***	
TIII	Small	Dark	Small	Medium	Yes	**	
TIV	Medium	Dark	Large	Big	No	***	Compact flowers
TV	Small	Medium	Small	Medium	Yes	*	Strong anthocyanin coloration of the whole plant
Reference type	Tall	Dark	-	-	-	*****	
*‘Finola selection’*							
FI	Tall	Dark	Medium	Big	No	*****	
FII	Medium	Medium	Medium	Big	No	***	
FIII	Medium	Light	Medium	Medium	Yes	*****	
FIV	Medium	Dark	Medium	Big	Yes	*****	
Reference type	Small	Medium	Small-medium	-	No	***	

Legend: Size: comparison between the height of plants within phenotypes in each variety; color of plants: light, medium, or dark green; branching: *—little branched, *****—highly branched, - no data available.

**Table 2:** Average essential oil (EO) content (mL/100 g air-dried flower) of the main components in the inflorescence and average composition (%) of essential oil. Groups (a, b, c, and d) were determined by analysis of variance (ANOVA) from different phenotypes of Carmagnola selected, Tiborszallasi, and Finola selection. The same letters present similarities between phenotypes, while different letters present differences between phenotypes according to components of essential oil. The mean ± standard deviation (SD) is reported.

Phenotype	CI	SD	CII	SD	TI	SD	TII	SD	TIII	SD	TIV	SD	TV	SD	FI	SD	FII	SD	FIII	SD	FIV	SD
Average EO content	0.23	0.10	0.53	0.16	0.58	0.23	0.39	0.04	0.52	0.15	0.64	0.22	0.56	0.17	2.75	0.33	3.11	0.23	2.82	0.41	2.59	0.38
α- Pinene	2.5 ^a,b^	1.7	11.6 ^c,d^	4.6	10.3 ^b,c,d^	3.3	7.4 ^a,b,c,d^	3.7	8.3 ^a,b,c,d^	5.2	11.9 ^d^	6.3	11.5 ^c,d^	12.2	3.7 ^a,b,c^	4.1	6.0 ^a,b,c,d^	3.4	3.5 ^a,b^	3.4	0.7 ^a^	0.4
β-Pinene	2.1 ^a,b^	0.9	7.0 ^c^	3.3	3.9 ^a,b^	0.4	3.3 ^a,b^	1.3	3.5 ^a,b^	1.9	4.5 ^a,b^	2.1	4.5 ^a,b^	3.6	2.3 ^a,b^	1.5	3.6 ^a,b^	0.9	2.8 ^a,b^	1.0	1.3 ^a^	0.7
Myrcene	10.5 ^a^	7.0	20.9 ^a,b,c^	13.0	29.9 ^c^	4.4	21.4 ^a,b,c^	8.9	25.2 ^b,c^	8.1	26.7 ^b,c^	7.2	24.9 ^b,c^	9.3	19.2 ^a,b,c^	12.3	21.7 ^a,b,c^	10.7	16.0 ^a,b^	7.4	16.1 ^a,b^	7.6
Limonene	2.6 ^a,b,c^	2.5	3.3 ^a,b,c^	2.6	0.6 ^a^	0.4	1.8 ^a,b^	3.1	1.0 ^a^	1.4	1.7 ^a,b^	3.0	1.2 ^a^	1.1	4.1 ^b,c^	0.9	4.4 ^c^	0.6	4.5 ^c^	1.6	5.2 ^c^	0.7
Terpinolene	2.9 ^a^	1.8	13.4 ^b,c^	8.3	2.7 ^a^	4.1	2.8 ^a^	3.7	2.6 ^a^	3.1	3.8 ^a,b^	5.1	4.2 ^a,b^	4.9	5.5 ^a,b^	7.1	10.6 ^a,b,c^	9.6	16.0 ^c^	9.1	7.9 ^a,b,c^	10.2
β-Caryophyllene	21.3 ^c^	6.9	10.5 ^a^	3.0	10.8 ^a^	4.4	16.1 ^a,b,c^	8.1	16.3 ^a,b,c^	4.4	11.9 ^a^	7.7	16.1 ^a,b,c^	7.9	19.7 ^b,c^	5.6	12.5 ^a,b^	3.6	13.6 ^a,b^	3.1	19.7 ^b,c^	6.9
α-Humulene	8.8 ^b^	2.8	4.5 ^a^	1.4	4.0 ^a^	2.2	5.6 ^a^	2.6	5.7 ^a^	1.8	4.1 ^a^	2.5	5.0 ^a^	2.4	6.4 ^a,b^	2.1	3.9 ^a^	1.1	4.2 ^a^	1.0	6.7 ^a,b^	2.7
Caryophyllene oxide	2.9 ^d^	0.8	1.1 ^b^	0.9	1.5 ^b,c^	0.4	2.6 ^d^	0.9	2.2 ^c,d^	0.8	1.6 ^b,c^	0.5	1.3 ^b^	0.5	0.3 ^a^	0.2	0.3 ^a^	0.1	0.3 ^a^	0.1	0.4 ^a^	0.2
β-Eudesmol	2.8 ^b^	1.9	0.9 ^a^	1.5	0.5 ^a^	0.6	0.8 ^a^	0.8	1.0 ^a^	1.5	0.8 ^a^	0.8	0.4 ^a^	0.4	1.3 ^a^	0.22	1.31 ^a^	0.24	1.09 ^a^	0.47	1.41 ^a^	0.24
α-Bisabolol	0.9 ^a^	0.3	0.4 ^a^	0.4	0.7 ^a^	0.6	0.4 ^a^	0.3	0.8 ^a^	0.8	0.6 ^a^	0.4	0.2 ^a^	0.1	4.8 ^d^	1.15	3.03 ^b^	1.67	3.37 ^b,c^	0.90	4.31 ^c,d^	0.26

**Table 3:** Average cannabinoid content (%) of Carmagnola selected, Tiborszallasi, and Finola selection. Groups (a, b, c, and d) were formed by analysis of variance (ANOVA) using different hemp phenotypes. The same letters present similarities between phenotypes, while different letters present differences between phenotypes. Values are presented as the mean ± standard deviation (SD).

	CI	SD	CII	SD	TI	SD	TII	SD	TIII	SD	TIV	SD	TV	SD	FI	SD	FII	SD	FIII	SD	FIV	SD
CBD	0.05 ^a^	0.04	0.10 ^a^	0.04	0.07 ^a^	0.03	0.04 ^a^	0.02	0.05 ^a^	0.02	0.07 ^a^	0.04	0.06 ^a^	0.03	0.60 ^b^	0.08	0.78 ^c^	0.12	0.61 ^b^	0.12	0.64 ^b^	0.19
CBD-A	1.70 ^a^	1.53	3.78 ^b^	0.94	3.22 ^b^	1.11	3.11 ^b^	0.56	2.65 ^a,b^	1.06	3.78 ^b^	0.62	2.93 ^b^	1.37	6.48 ^c^	0.35	6.41 ^c^	0.35	6.36 ^c^	0.25	6.59 ^c^	0.24
CBG	0.04 ^b,c^	0.02	0.04 ^b,c^	0.01	0.01 ^a^	0.01	0.01 ^a^	0.01	0.01 ^a^	0.01	0.02 ^a,b^	0.02	0.02 ^a^	0.01	0.05 ^c,d^	0.01	0.05 ^c,d^	0.01	0.06 ^d^	0.01	0.06 ^d^	0.02
CBG-A	1.62 ^b^	2.39	0.44 ^a^	0.24	0.25 ^a^	0.08	0.16 ^a^	0.05	0.19 ^a^	0.06	0.22 ^a^	0.07	0.18 ^a^	0.07	1.00 ^a,b^	0.37	0.63 ^a,b^	0.16	0.91 ^a,b^	0.23	1.11 ^a,b^	0.26
Δ-9-THC	0.05 ^a,b,c^	0.09	0.04 ^a,b,c^	0.05	0.04 ^a,b,c^	0.03	0.01 ^a^	0.01	0.02 ^a,b^	0.02	0.01 ^a^	0.01	0.06 ^b,c,d^	0.05	0.08 ^c,d^	0.02	0.11 ^d^	0.02	0.08 ^c,d^	0.01	0.08 ^c,d^	0.02
Δ-9-THC-A	0.91 ^a,b^	1.81	0.75 ^a,b^	1.26	0.71 ^a,b^	0.72	0.14 ^a^	0.03	0.53 ^a,b^	0.54	0.18 ^a^	0.04	1.39 ^b^	1.23	0.50 ^a,b^	0.08	0.46 ^a,b^	0.07	0.47 ^a,b^	0.06	0.51 ^a,b^	0.05
CBC	0.01 ^a,b^	0.00	0.02 ^a^	0.01	0.01 ^a,b^	0.01	0.00 ^a^	0.00	0.01 ^a,b^	0.01	0.01 ^a,b^	0.01	0.01 ^a,b^	0.00	0.05 ^c^	0.01	0.06 ^d^	0.01	0.05 ^c^	0.01	0.05 ^c^	0.01
CBC-A	0.20 ^a^	0.06	0.27 ^a^	0.09	0.34 ^a^	0.21	0.24 ^a^	0.10	0.25 ^a^	0.07	0.34 ^a^	0.16	0.30 ^a^	0.16	0.62 ^b^	0.10	0.59 ^b^	0.09	0.64 ^b^	0.10	0.64 ^b^	0.10

## Data Availability

Not applicable.

## Supplementary Materials

The following are available online at https://www.mdpi.com/article/10.3390/plants10050966/s1, Figure S1: Principal component analysis (PCA) plot for three different hemp populations (Carmagnola selected, Tiborszallasi, and Finola selection) according to 29 components of essential oil, made on first two PC scores (PC1 explained 24.43%, and PC2 explained 17.53%), with a total variance of 41.96%, Figure S2: PCA plot for all three populations (Carmagnola selected, Tiborszallasi, and Finola selection) according to all cannabinoids made on first two PC scores (PC1 explained 50.71%, and PC2 explained 17.00%) with a total variance of 67.71%, Figure S3: PCA plot for all analyzed samples of all populations according to components of essential oil and cannabinoids made on the first two PC scores (PC1 explained 27.38%, and PC2 explained 16.20%) with a total variance of 43.58%, Figure S4: Phenotype CI: Plants of CI were light green with large primary leaves, Figure S5: Phenotype CII: Plants of the CII phenotype were dark green with narrow leaflets and anthocyanin coloration on the leaf petiole. Most plants had more lateral branches, Figure S6: Phenotype TI: TI plants were taller compared to the other phenotypes with lateral branches and smaller inflorescences, Figure S7: Phenotype TII: TII plants were dark green with anthocyanin coloration of leaf petioles on the primary and secondary branches, Figure S8: Phenotype TIII: TIII plants were also dark green, smaller than other phenotypes, with less lateral branches, anthocyanin coloration of the leaf petioles, and pink bracts, Figure S9: Phenotype TIV: TIV plants were dark green with big compact inflorescences and thin leaflets around the inflorescences, Figure S10: TV plants were small and had strong anthocyanin coloration of upper and lower leaves, inflorescences, and leaf petioles, Figure S11: Phenotype FI: Plants of the FI phenotype were dark green with compact growth and taller than other phenotypes of FS. Plants had long side branches with longer and larger leaves and higher main inflorescences, Figure S12: Phenotype FII: FII plants were medium green with shorter side branches and compact inflorescences surrounded by many leaflets, Figure S13: Phenotype FIII: Plants from the FIII phenotype were light green with long side branches and a wide angle between them. Leaves had medium anthocyanin-colored edges, and inflorescences had green leaflets and were not compact, Figure S14: Phenotype FIV: FIV plants were dark green with long inflorescences, and long, upright side branches. Leaf petioles had strong anthocyanin coloration, Table S1: Average essential oil (EO) content (mL/100 g of air-dried hemp) in the inflorescences, average composition (%) of essential oil, and groups (a, b, c, and d) from statistical analysis performed by analysis of variance (ANOVA) from different phenotypes of Carmagnola selected, Tiborszallasi, and Finola selection. The same letters present similarities between phenotypes, while different letters present differences between phenotypes according to components of essential oil. The mean ± standard deviation (SD) is reported, Table S2: Chemical structures of components of essential oil and cannabinoids described in the research.
