# Supplementary material for: Metabolomic Analysis of Cannabinoid and Essential Oil Profiles in Different Hemp (Cannabis sativa L.) Phenotypes
Source: Plants (Basel). 2021 May 12;10(5):966. doi: 10.3390/plants10050966 (PMC8151046; doi:10.3390/plants10050966)
Supplement: Supplementary file 1 [file plants-10-00966-s001.zip › plants-1193044-supplementary.pdf]

SUPPLEMENTARY MATERIAL

Metabolomic analysis of cannabinoid and essential oil profile in different hemp (*Cannabis sativa* L.) phenotypes

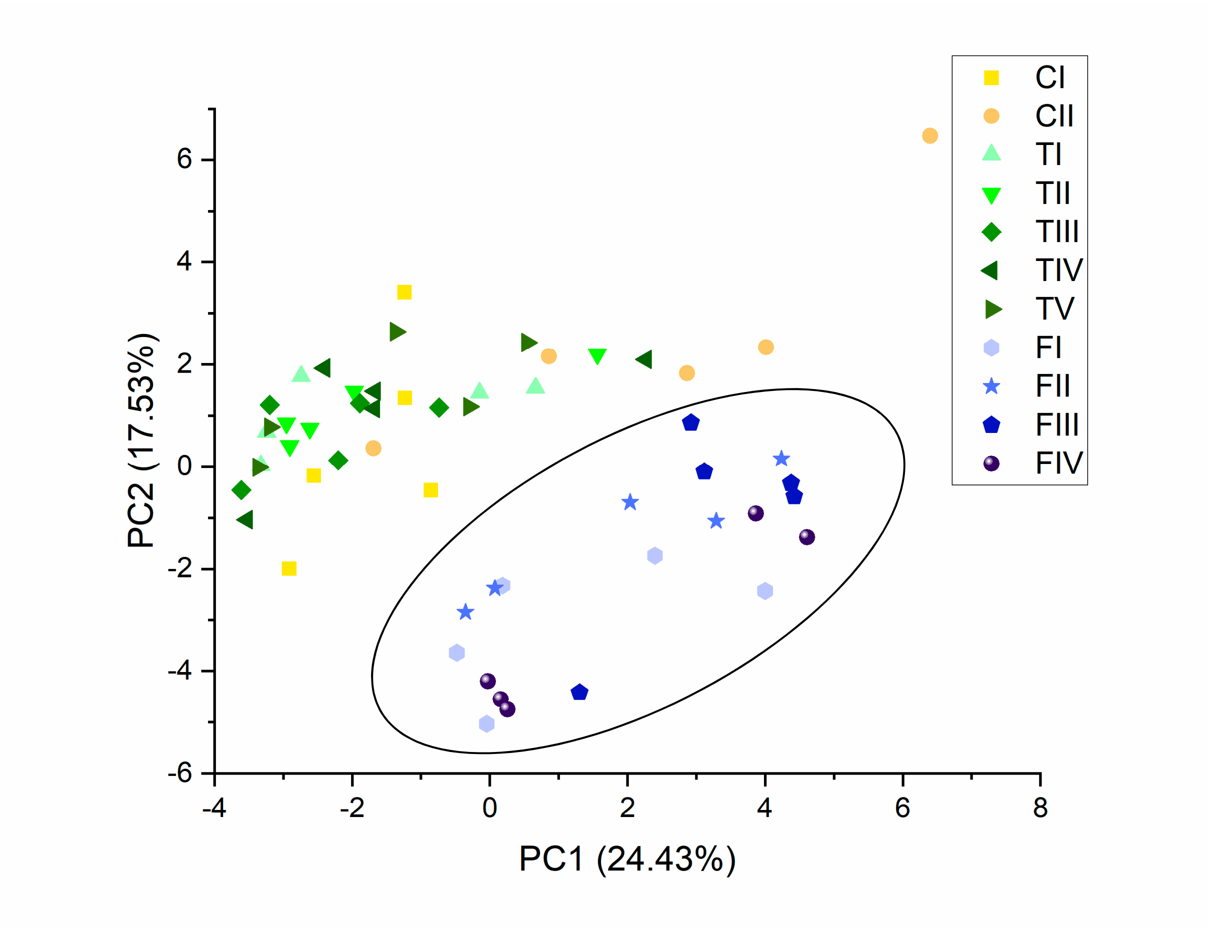

Figure S1. Principal component analysis (PCA) plot for three different hemp populations (Carmagnola selected, Tiborszallasi, and Finola selection) according to 29 components of essential oil, made on first two PC scores (PC1 explained 24.43% and PC2 explained 17.53%), with total variance of 41.96%.

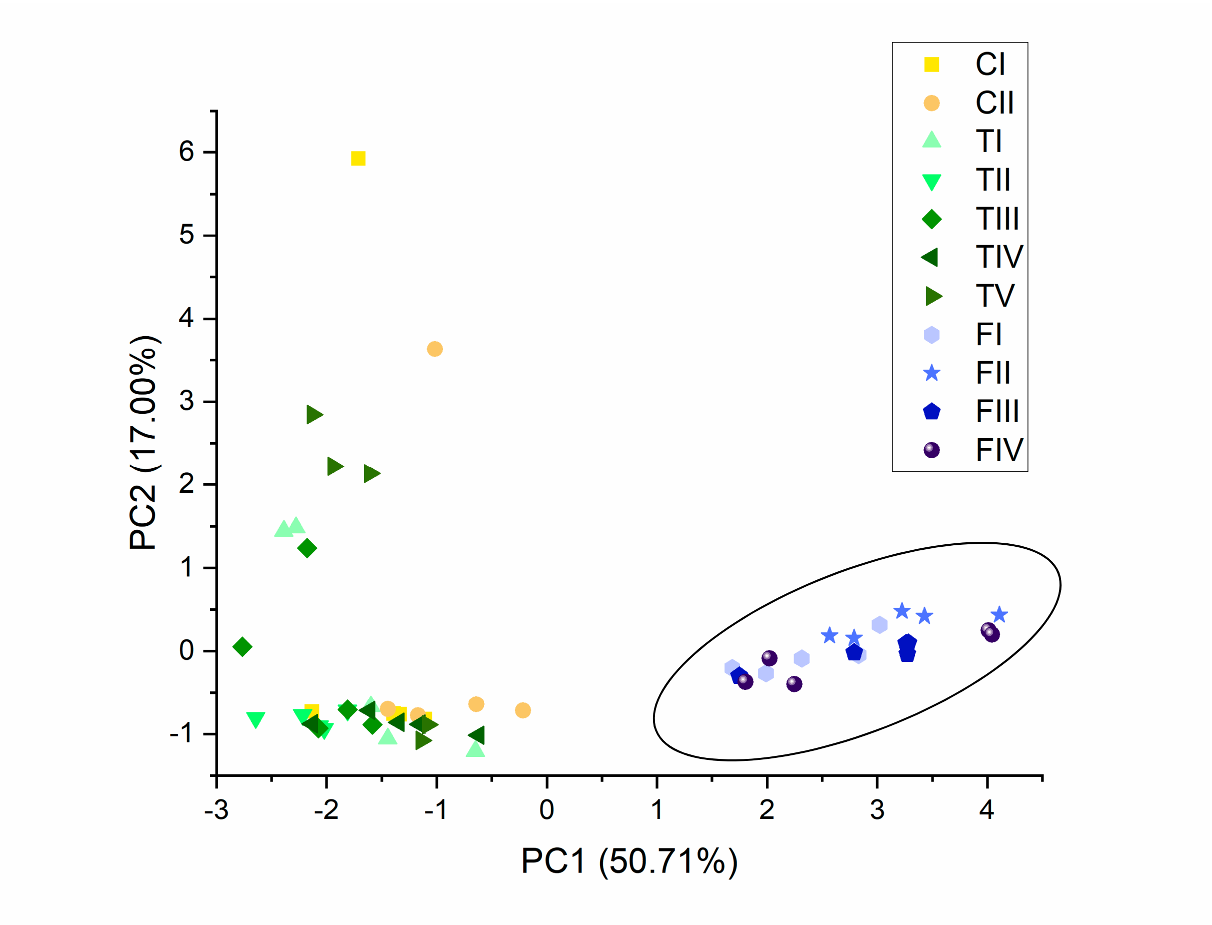

Figure S2. PCA plot for all three populations (Carmagnola selected, Tiborszallasi and Finola selection) according to all cannabinoids made on first two PC scores (PC1 explained 50.71% and PC2 explained 17.00%) with total variance of 67.71%.

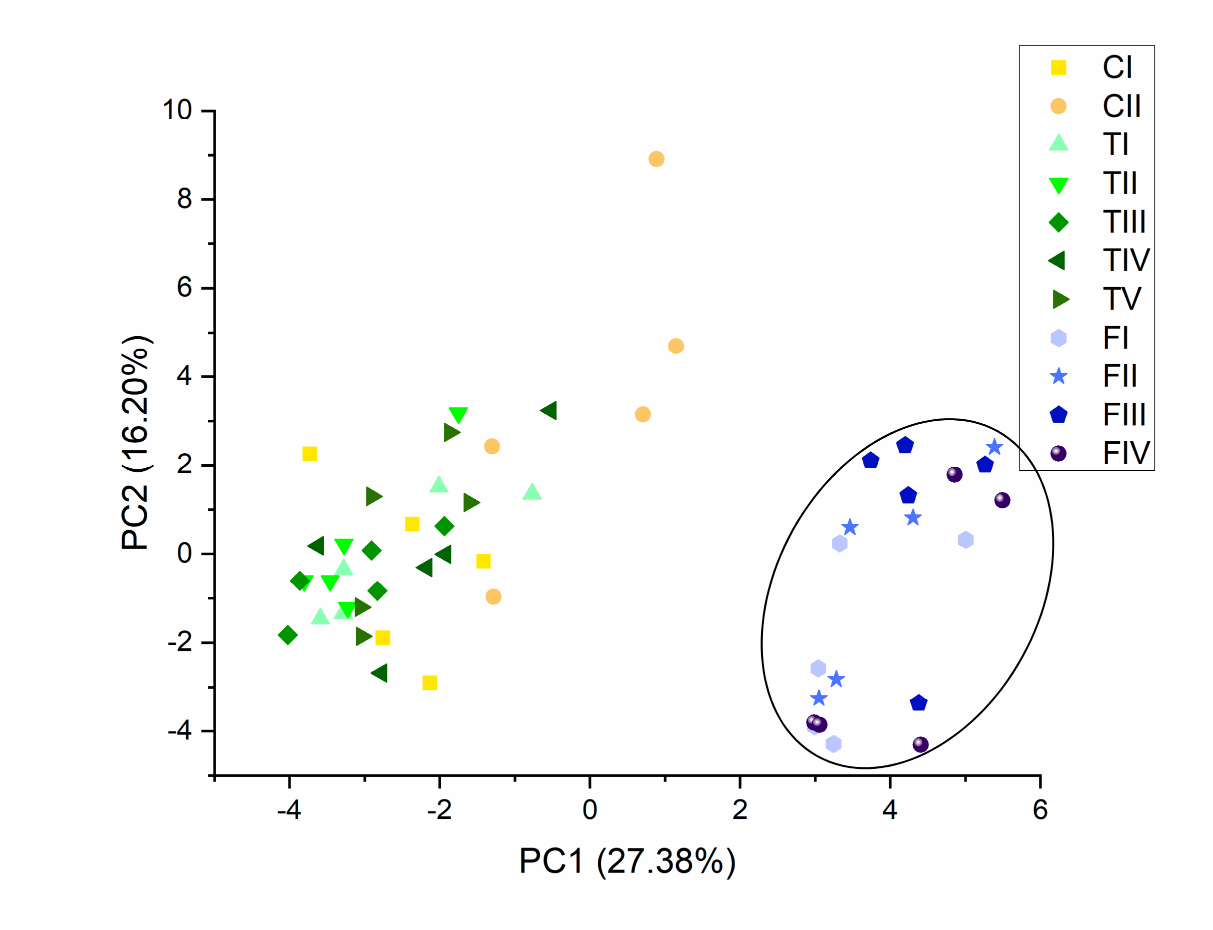

Figure S3. PCA plot for all analyzed samples of all populations according to components of essential oil and cannabinoids made on the first two PC scores (PC1 explained 27.38% and PC2 explained 16.20%) with a total variance of 43.58%.

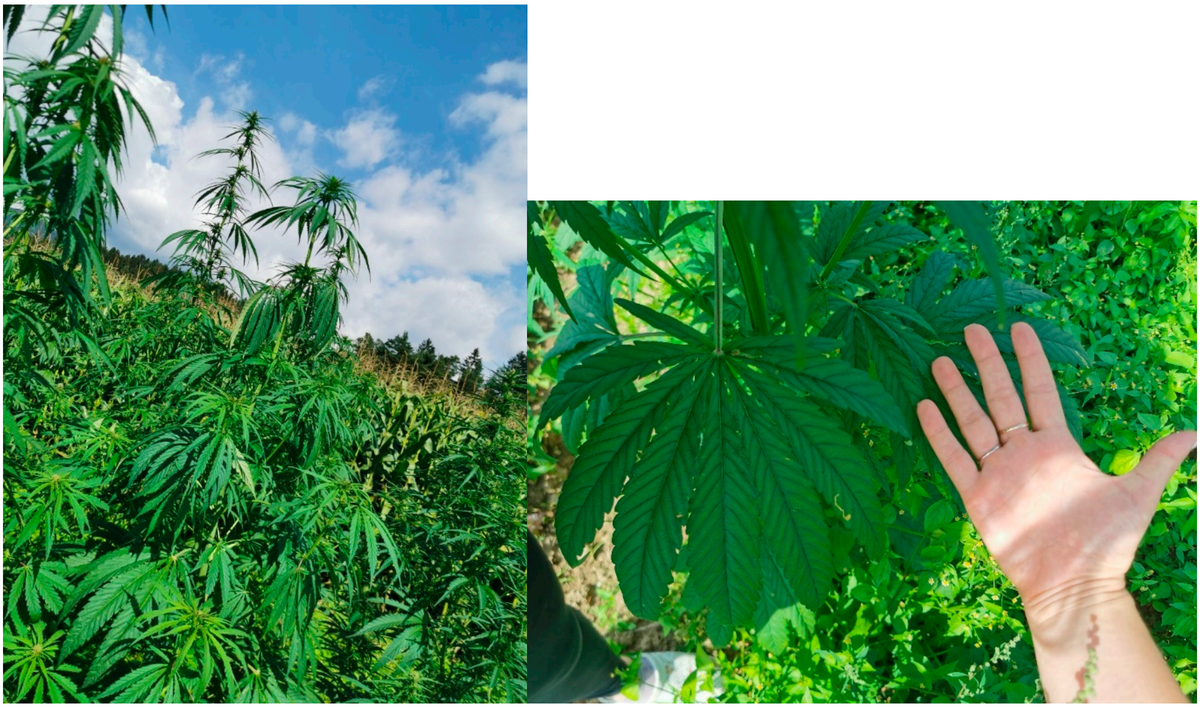

Figure S4. Phenotype CI. Plants of CI were light green with large primary leaves.

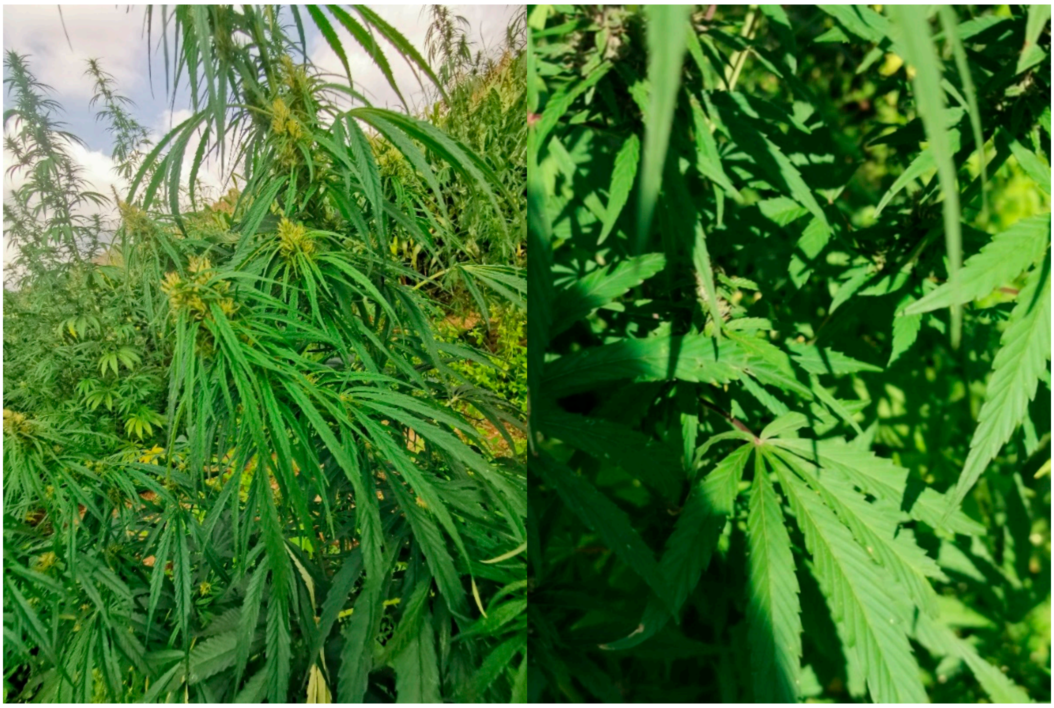

Figure S5. Phenotype CII. Plants of the CII phenotype were dark green with narrow leaflets and anthocyanin coloration on the leaf petiole. Most plants had more lateral branches.

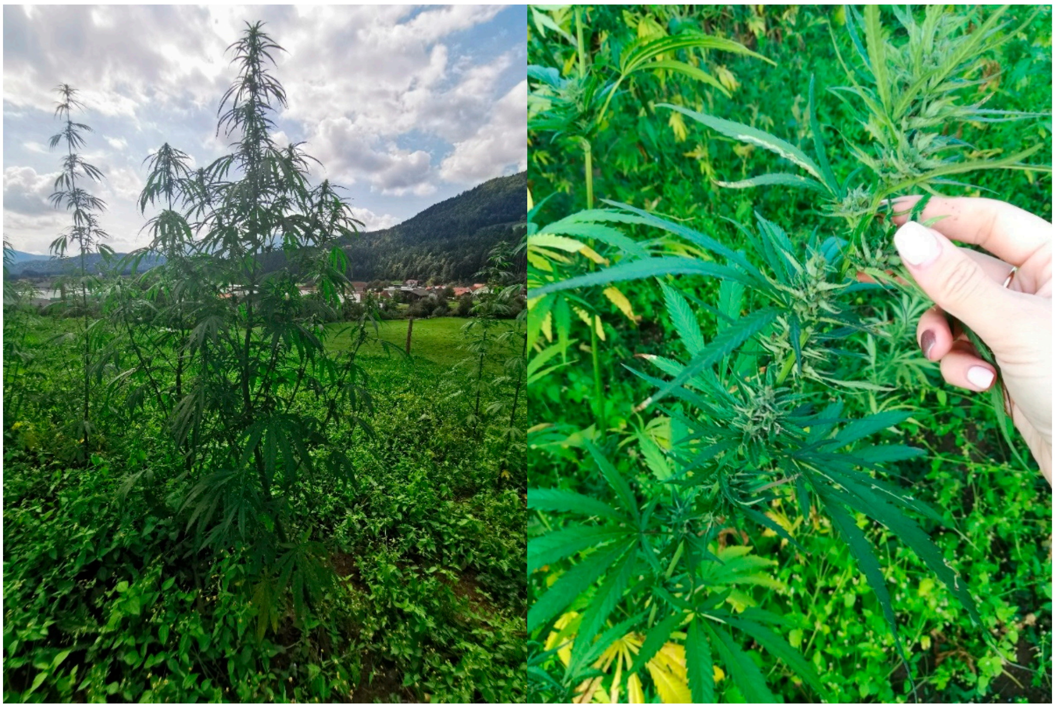

Figure S6. Phenotype TI. TI plants were taller compared to the other phenotypes with lateral branches and smaller inflorescences.

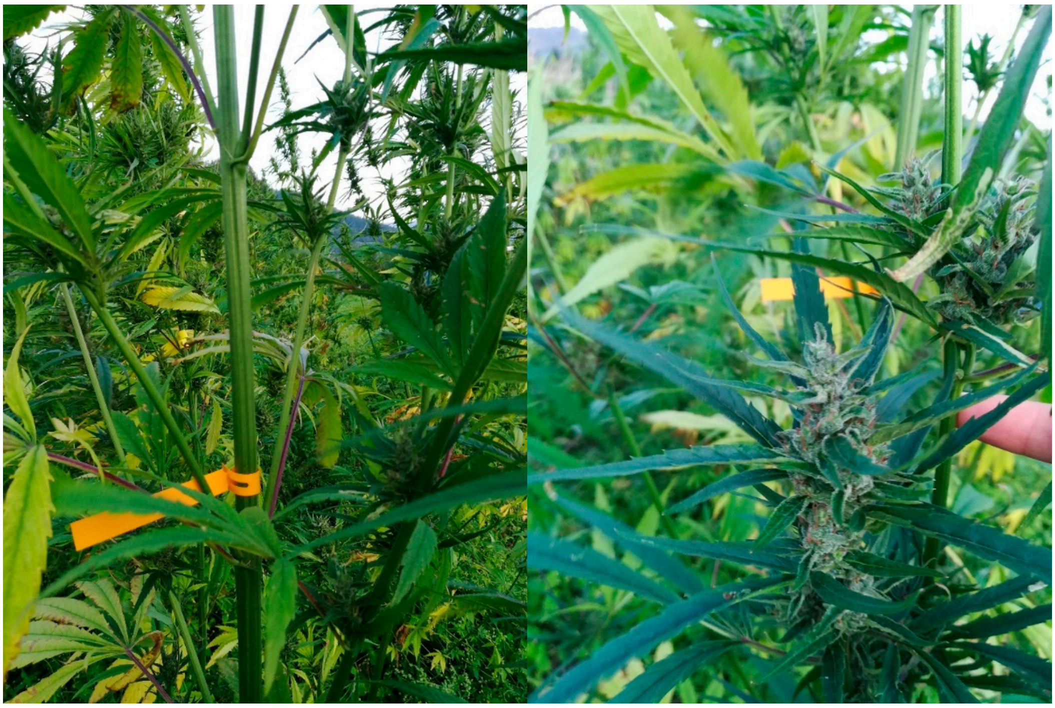

Figure S7. Phenotype TII. TII plants were dark green with anthocyanin coloration of leaf petioles on the primary and secondary branches.

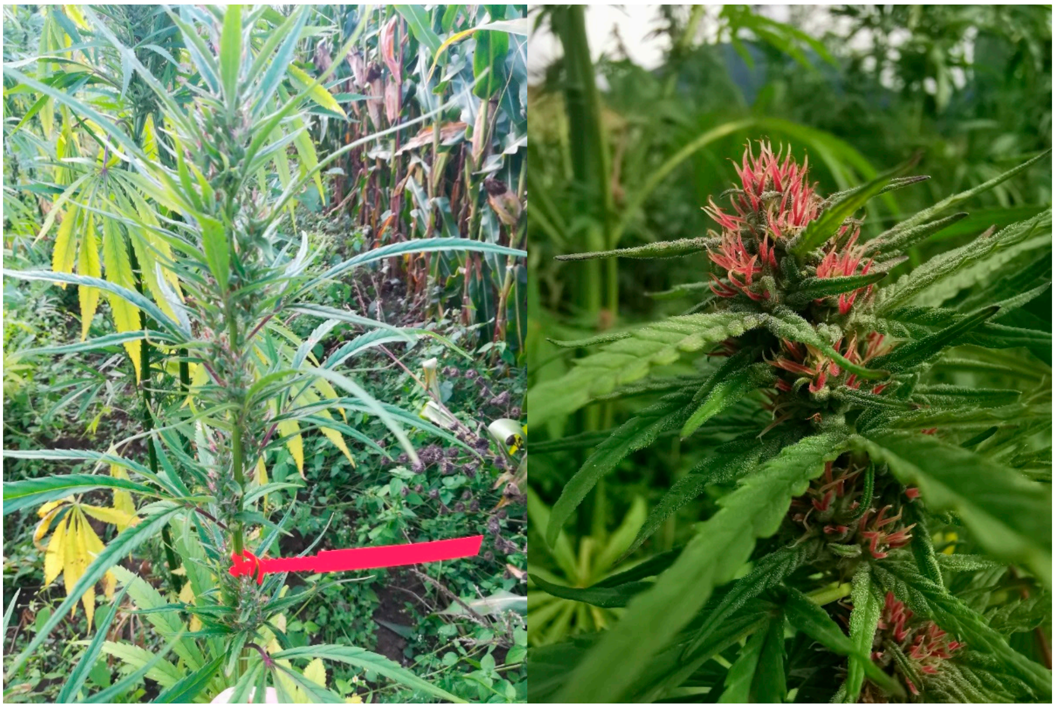

Figure S8. Phenotype TIII. TIII plants were also dark green, smaller than other phenotypes, with less lateral branches, anthocyanin coloration of the leaf petioles, and pink bracts.

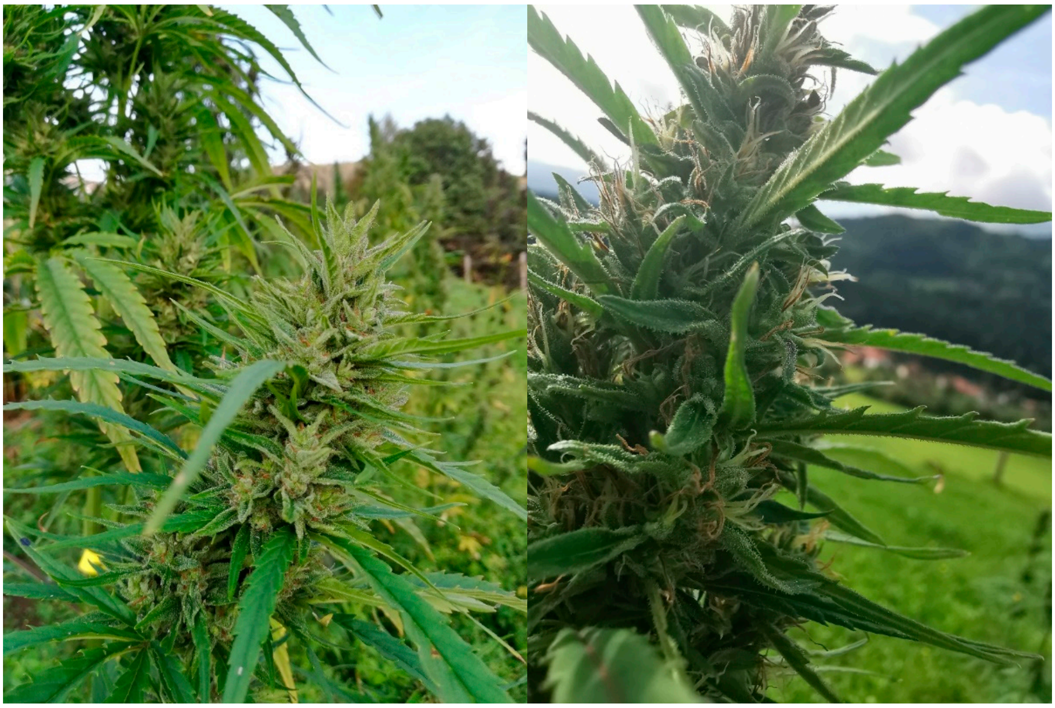

Figure S9. Phenotype TIV. TIV plants were dark green with big compact inflorescences and thin leaflets around the inflorescences.

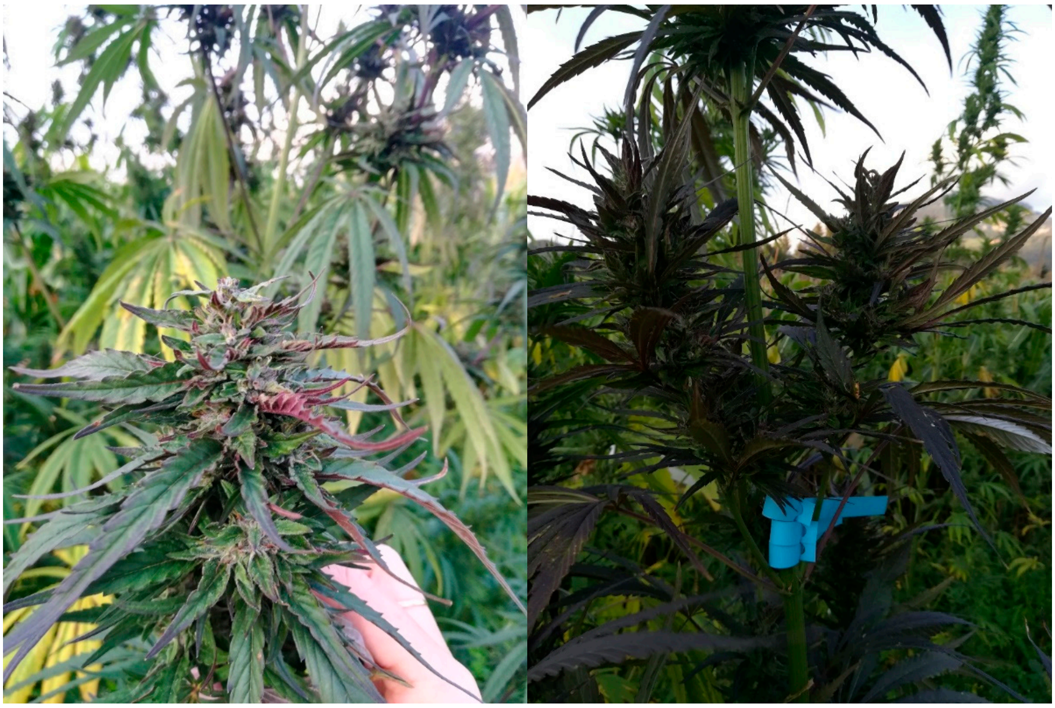

Figure S10. TV plants were small and had strong anthocyanin coloration of upper and lower leaves, inflorescences, and leaf petioles.

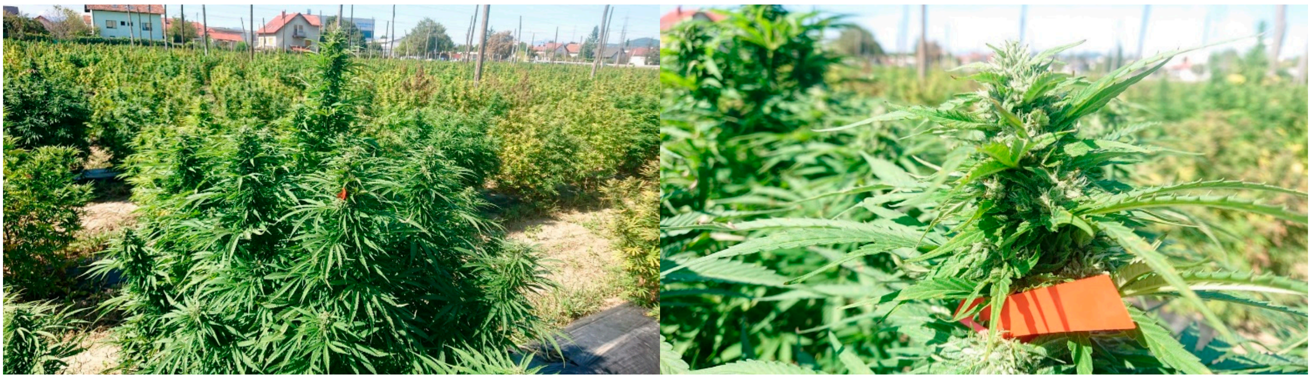

Figure S11. Phenotype FI. Plants of the FI phenotype were dark green with compact growth and taller than other phenotypes of FS. Plants had long side branches with longer and larger leaves and higher main inflorescences.

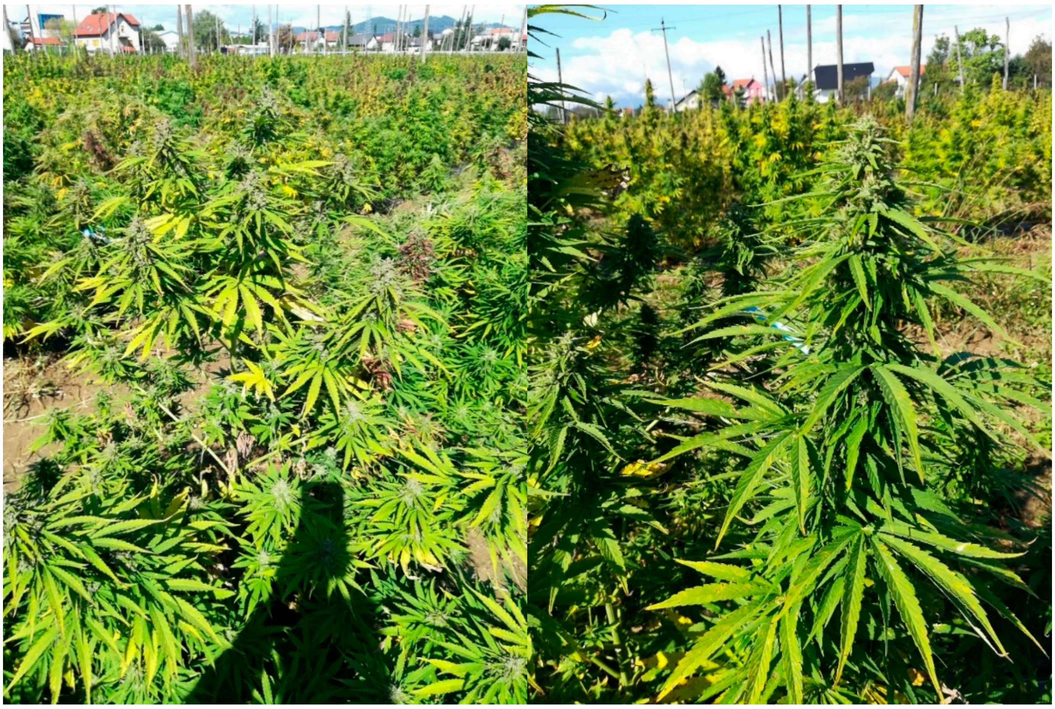

Figure S12. Phenotype FII. FII plants were medium green with shorter side branches and compact inflorescences surrounded by many leaflets.

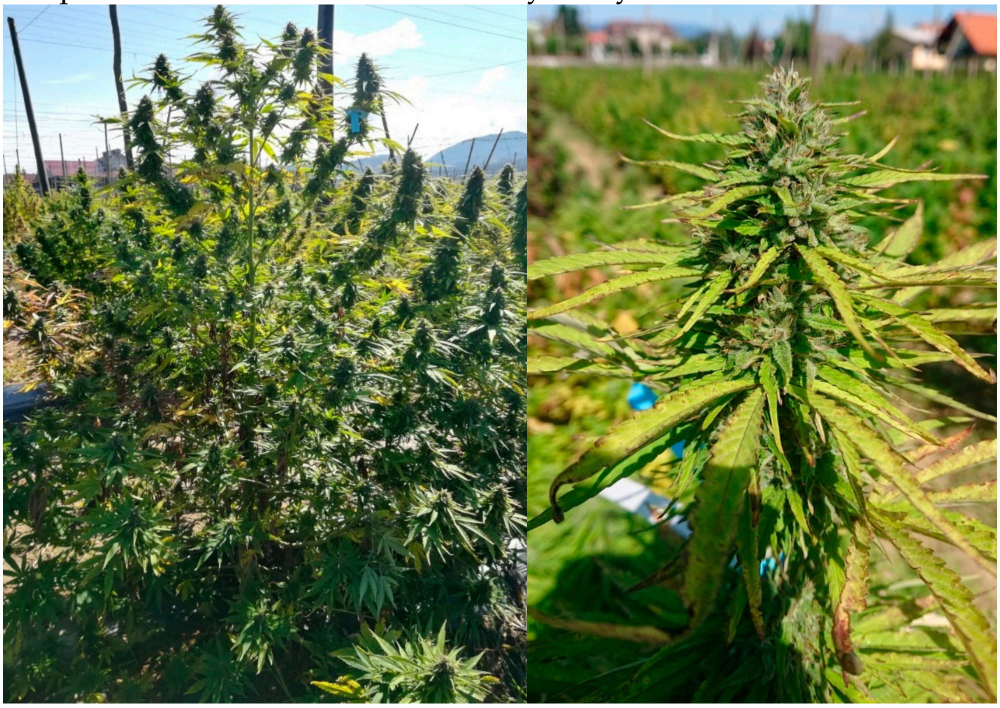

Figure S13. Phenotype FIII. Plants from the FIII phenotype were light green with long side branches and a wide angle between them. Leaves had medium anthocyanin-colored edges, and inflorescences had green leaflets and were not compact.

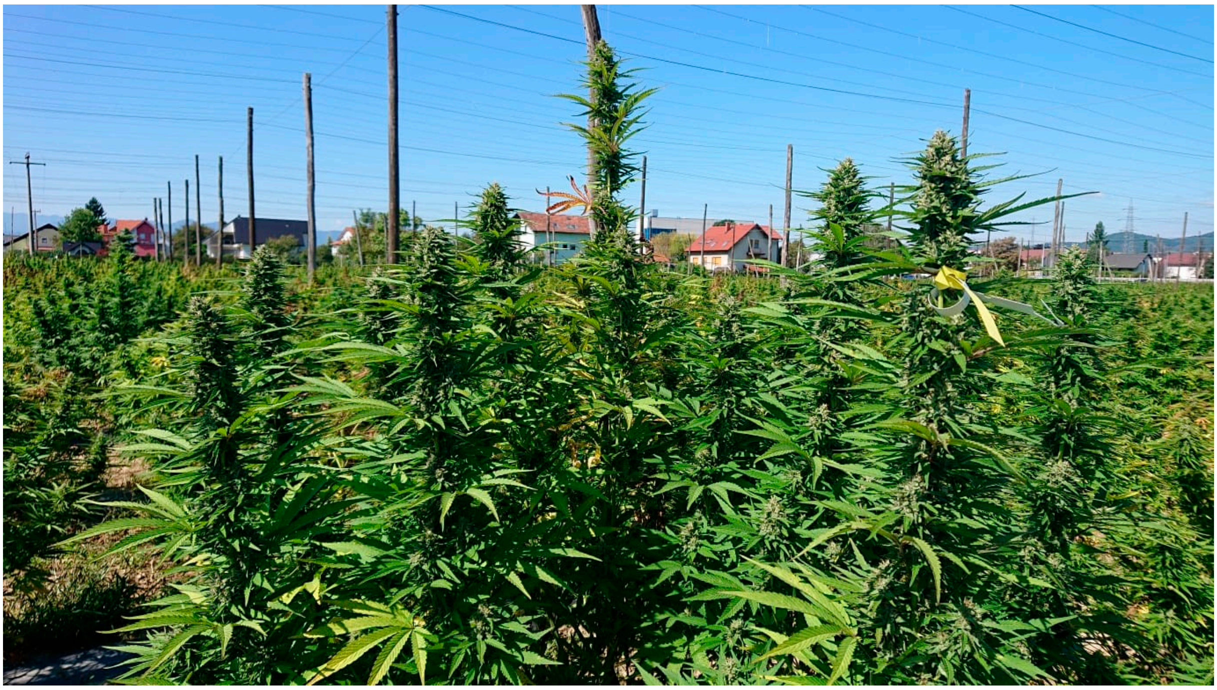

Figure S14. Phenotype FIV. FIV plants were dark green with long inflorescences, and long, upright side branches. Leaf petioles had strong anthocyanin coloration.

Table S1. Average essential oil (EO) content (mL/100 g of air dry hemp) in the inflorescences, average composition (%) of essential oil and groups (a, b, c, d) from statistical analysis performed by analysis of variance (ANOVA) from different phenotypes of Carmagnola selected, Tiborszallasi, and Finola selection. The same letters present similarities between phenotypes, while different letters present differences between phenotypes according to components of essential oil. The mean ± standard deviation (SD) is reported.

**Phenotype**	**CI**	**SD**	**CII**	**SD**	**TI**	**SD**	**TII**	**SD**	**TIII**	**SD**	**TIV**	**SD**
Average EO content	0.23	*0.10*	0.53	*0.16*	0.58	*0.23*	0.39	*0.04*	0.52	*0.15*	0.64	*0.22*
α- Pinene	2.5^{ab}	*1.7*	11.6^{cd}	*4.6*	10.3^{bcd}	*3.3*	7.4^{abcd}	*3.7*	8.3^{abcd}	*5.2*	11.9^{d}	*6.3*
Camphene	0.1^{a}	*0.04*	0.3^{ab}	*0.1*	0.2^{ab}	*0.1*	0.2^{ab}	*0.1*	0.2^{ab}	*0.1*	0.2^{ab}	*0.1*
β-Pinene	2.1^{ab}	*0.9*	7.0^{c}	*3.3*	3.9^{ab}	*0.4*	3.3^{ab}	*1.3*	3.5^{ab}	*1.9*	4.5^{ab}	*2.1*
Myrcene	10.5^{a}	*7.0*	20.9^{abc}	*13.0*	29.9^{c}	*4.4*	21.4^{abc}	*8.9*	25.2^{bc}	*8.1*	26.7^{bc}	*7.2*
3-Carene	0.2^{a}	*0.2*	0.8^{c}	*0.5*	0.2^{ab}	*0.2*	0.3^{ab}	*0.2*	0.3^{ab}	*0.2*	0.3^{ab}	*0.3*
α- Terpinene	0.2^{ab}	*0.1*	0.6^{bc}	*0.3*	0.1^{a}	*0.2*	0.2^{a}	*0.1*	0.1^{a}	*0.1*	0.2^{ab}	*0.2*
p-Cymene	0.1	*0.0*	0.1	*0.0*	0.1	*0.1*	0.0	*0.0*	0.0	*0.0*	0.0	*0.0*
Limonene	2.6^{abc}	*2.5*	3.3^{abc}	*2.6*	0.6^{a}	*0.4*	1.8^{ab}	*3.1*	1.0^{a}	*1.4*	1.7^{ab}	*3.0*
γ-Terpinene	0.3^{abc}	*0.2*	0.6^{c}	*0.3*	0.2^{ab}	*0.2*	0.2^{ab}	*0.1*	0.1^{a}	*0.1*	0.1^{ab}	*0.2*
Fenchone	0.0	*0.0*	0.1	*0.0*	0.0	*0.0*	0.1	*0.1*	0.0	*0.00*	0.0	*0.0*
Terpinolene	2.9^{a}	*1.8*	13.4^{bc}	*8.3*	2.7^{a}	*4.1*	2.8^{a}	*3.7*	2.6^{a}	*3.1*	3.8^{ab}	*5.1*
Linalool	0.4^{a}	*0.3*	0.4^{a}	*0.3*	0.1^{a}	*0.1*	0.2^{a}	*0.4*	0.1^{a}	*0.1*	0.2^{a}	*0.3*

Camphor	0.0	0.0	0.1	0.0	0.0	0.0	0.0	0.0	0.0	0.0	0.0	0.0
Isoborneol	0.1	0.0	0.1	0.1	0.1	0.0	0.1	0.0	0.1	0.0	0.1	0.0
Borneol	0.1	0.1	0.2	0.0	0.1	0.1	0.1	0.1	0.1	0.0	0.1	0.1
Menthol	0.2^{ab}	0.1	0.4^{b}	0.2	0.1^{a}	0.1	0.1^{a}	0.1	0.1^{a}	0.1	0.1^{a}	0.1
α-Terpineol	0.1^{a}	0.1	0.2^{a}	0.2	0.0^{a}	0.0	0.1^{a}	0.0	0.0^{a}	0.0	0.1^{a}	0.1
β-Citronellol	0.0	0.0	0.0	0.0	0.1	0.1	0.1	0.1	0.0	0.0	0.0	0.1
Neryl acetate	0.0	0.0	0.0	0.0	0.0	0.0	0.0	0.0	0.0	0.0	0.0	0.0
Geranyl acetate	0.1^{a}	0.1	0.1^{ab}	0.1	0.1^{ab}	0.2	0.1^{a}	0.0	0.0^{a}	0.0	0.0^{a}	0.0
α-Cedrene	0.2	0.1	0.1	0.1	0.1	0.0	0.1	0.0	0.1	0.0	0.1	0.0
β-Caryophyllene	21.3^{c}	6.9	10.5^{a}	3.0	10.8^{a}	4.4	16.1^{abc}	8.1	16.3^{abc}	4.4	11.9^{a}	7.7
α-Humulene	8.8^{b}	2.8	4.5^{a}	1.4	4.0^{a}	2.2	5.6^{a}	2.6	5.7^{a}	1.8	4.1^{a}	2.5
cis-Nerolidol	0.4^{c}	0.3	0.1^{a}	0.1	0.3^{bc}	0.2	0.3^{bc}	0.2	0.2^{ab}	0.1	0.2^{ab}	0.1
Geranyl isobutyrate	0.8^{c}	0.6	0.2^{ab}	0.1	0.4^{abc}	0.2	0.4^{abc}	0.2	0.4^{abc}	0.1	0.4^{abc}	0.3
Caryophyllene oxide	2.9^{d}	0.8	1.1^{b}	0.9	1.5^{bc}	0.4	2.6^{d}	0.9	2.2^{cd}	0.8	1.6^{bc}	0.5
β-Eudesmol	2.8^{b}	1.9	0.9^{a}	1.5	0.5^{a}	0.6	0.8^{a}	0.8	1.0^{a}	1.5	0.8^{a}	0.8
α-Bisabolol	0.9^{a}	0.3	0.4^{a}	0.4	0.7^{a}	0.6	0.4^{a}	0.3	0.8^{a}	0.8	0.6^{a}	0.4
Phytol	0.7^{b}	1.2	0.1^{a}	0.0	0.1^{a}	0.0	0.1^{a}	0.1	0.1^{a}	0.1	0.1^{a}	0.0

continued

**Phenotype**	**TV**	**SD**	**FI**	**SD**	**FII**	**SD**	**FIII**	**SD**	**FIV**	**SD**
Average EO content	0.56	0.17	2.75	0.33	3.11	0.23	2.82	0.41	2.59	0.38
α- Pinene	11.5^{cd}	12.2	3.7^{abc}	4.1	6.0^{abcd}	3.4	3.5^{ab}	3.4	0.7^{a}	0.4
Camphene	0.2^{ab}	0.2	0.1^{ab}	0.1	0.2^{ab}	0.1	0.1^{ab}	0.1	0.1^{a}	0.0
β-Pinene	4.5^{ab}	3.6	2. 3^{ab}	1.5	3.6^{ab}	0.9	2.8^{ab}	1.0	1.3^{a}	0.7
Myrcene	24.9^{bc}	9.3	19.2^{abc}	12.3	21.7^{abc}	10.7	16.0^{ab}	7.4	16.1^{ab}	7.6
3-Carene	0.5^{abc}	0.3	0.3^{ab}	0.3	0.4^{abc}	0.4	0.6^{bc}	0.3	0.3^{ab}	0.4
α- Terpinene	0.2^{ab}	0.2	0.3^{abc}	0.3	0.4^{abc}	0.4	0.6^{c}	0.3	0.3^{abc}	0.4
p-Cymene	0.0	0.0	0.0	0.0	0.0	0.0	0.0	0.0	0.1	0.1
Limonene	1.2^{a}	1.1	4.1^{bc}	0.9	4.4^{c}	0.6	4.5^{c}	1.6	5.2^{c}	0.7
γ-Terpinene	0.2^{ab}	0.2	0.2^{ab}	0.3	0.3^{abc}	0.3	0.5^{bc}	0.2	0.3^{abc}	0.3
Fenchone	0.0	0.0	0.1	0.0	0.1	0.1	0.2	0.2	0.1	0.0
Terpinolene	4.2^{ab}	4.9	5.5^{ab}	7.1	10.6^{abc}	9.6	16.0^{c}	9.1	7.9^{abc}	10.2
Linalool	0.1^{a}	0.2	0.4^{a}	0.1	0.4^{a}	0.1	0.3^{a}	0.2	0.4^{a}	0.2
Camphor	0.0	0.0	0.0	0.0	0.0	0.0	0.1	0.0	0.0	0.0
Isoborneol	0.1	0.0	0.0	0.0	0.0	0.0	0.0	0.0	0.0	0.0
Borneol	0.1	0.1	0.2	0.0	0.2	0.0	0.2	0.1	0.2	0.0
Menthol	0.2^{a}	0.1	0.1^{a}	0.2	0.2^{a}	0.1	0.3^{ab}	0.2	0.2^{ab}	0.2
α-Terpineol	0.0^{a}	0.0	1.0^{bc}	0.3	1.0^{c}	0.3	0.6^{b}	0.6	1.0^{c}	0.4
β-Citronellol	0.0	0.0	0.0	0.1	0.1	0.1	0.1	0.1	0.0	0.0

Neryl acetate	0.0	*0.0*	0.0	*0.0*	0.0	*0.0*	0.0	*0.0*	0.0	*0.0*
Geranyl acetate	0.1^{a}	*0.1*	0.2^{b}	*0.1*	0.1^{ab}	*0.1*	0.2^{ab}	*0.1*	0.3^{a}	*0.1*
α-Cedrene	0.1	*0.0*	0.0	*0.0*	0.0	*0.0*	0.0	*0.0*	0.0	*0.0*
β-Caryophyllene	16.1^{abc}	*7.9*	19.7^{bc}	*5.6*	12.5^{ab}	*3.6*	13.6^{ab}	*3.1*	19.7^{bc}	*6.9*
α-Humulene	5.0^{a}	*2.4*	6.4^{ab}	*2.1*	3.9^{a}	*1.1*	4.2^{a}	*1.0*	6.7^{ab}	*2.7*
cis-Nerolidol	0.3^{bc}	*0.2*	0.0^{a}	*0.0*	0.0^{a}	*0.0*	0.0^{a}	*0.0*	0.0^{a}	*0.0*
Geranyl isobutyrate	0.5^{bc}	*0.3*	0.1^{ab}	*0.1*	0.2^{ab}	*0.3*	0.3^{ab}	*0.3*	0.0^{a}	*0.0*
Caryophyllene oxide	1.3^{b}	*0.5*	0.3^{a}	*0.2*	0.3^{a}	*0.1*	0.3^{a}	*0.1*	0.4^{a}	*0.2*
β-Eudesmol	0.4^{a}	*0.4*	1.3^{a}	*0.22*	1.31^{a}	*0.24*	1.09^{a}	*0.47*	1.41^{a}	*0.24*
α-Bisabolol	0.2^{a}	*0.1*	4.8^{d}	*1.15*	3.03^{b}	*1.67*	3.37^{bc}	*0.90*	4.31^{cd}	*0.26*
Phytol	0.1^{a}	*0.1*	0.0^{a}	*0.0*	0.0^{a}	*0.0*	0.0^{a}	*0.0*	0.0^{a}	*0.0*

Table S2: Chemical structures of components of essential oil and cannabinoids described in the research.

**Components of essential oil**	**Chemical structure**
α- Pinene[1]	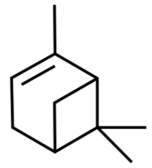
Camphene[1]	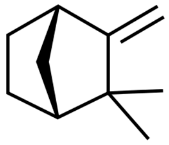
β-Pinene[1]	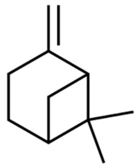
Myrcene[1]	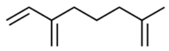
3-Carene[1]	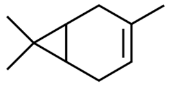

α- Terpinene[1]	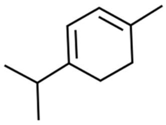
p-Cymene[1]	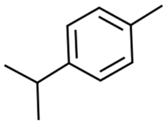
Limonene[1]	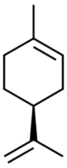
γ-Terpinene[1]	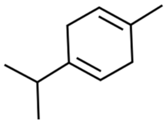
Fenchone[1]	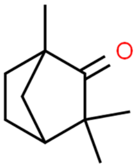
Terpinolene[1]	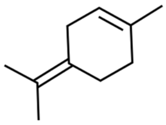
Linalool[1]	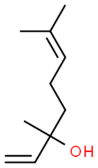
Camphor[1]	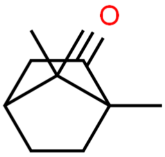
Isoborneol[2]	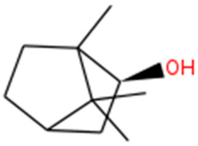

Borneol[1]	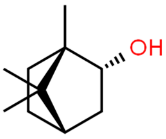
Menthol[1]	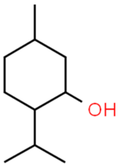
α-Terpineol[1]	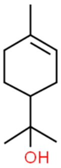
β-Citronellol[1]	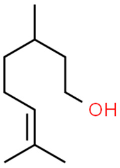
Neryl acetate[1]	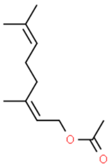
Geranyl acetate[3]	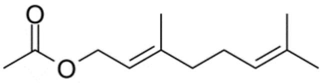
α-Cedrene[1]	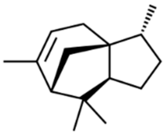
β-Caryophyllene[1]	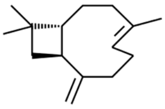
α-Humulene[1]	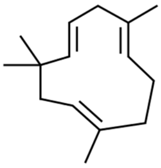

cis-Nerolidol[1]	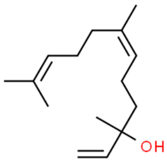
Geranyl isobutyrate[1]	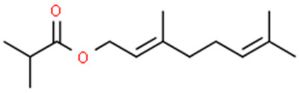
Caryophyllene oxide[1]	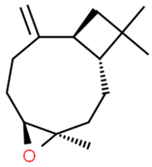
β-Eudesmol[1]	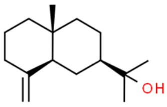
α-Bisabolol[1]	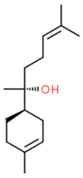
Phytol[1]	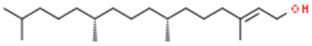

continued

**Cannabinoids**	**Chemical structure**
CBD[1]	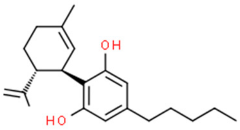
CBD-A[1]	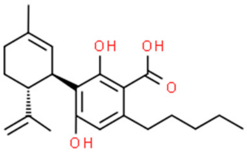
CBG[1]	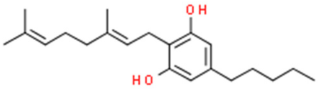

CBG-A[1]

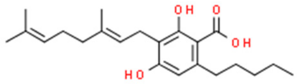

Δ-9-THC[1]

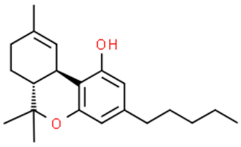

Δ-9-THC-A[1]

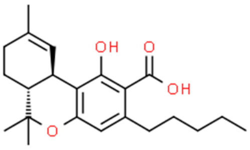

Δ-8-THC[1]

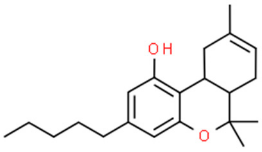

CBN[1]

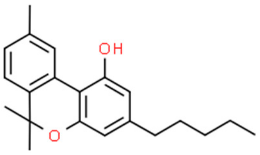

CBC[1]

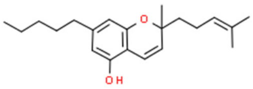

CBC-A[1]

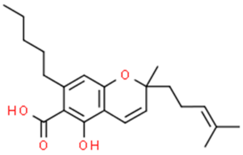

1 http://www.chemspider.com/

2 https://www.chemeo.com/

3 https://www.medchemexpress.com/
